# MutT homologue 1 (MTH1) catalyzes the hydrolysis of mutagenic O6-methyl-dGTP

**DOI:** 10.1093/nar/gky896

**Published:** 2018-10-10

**Authors:** Ann-Sofie Jemth, Robert Gustafsson, Lars Bräutigam, Linda Henriksson, Karl S A Vallin, Antonio Sarno, Ingrid Almlöf, Evert Homan, Azita Rasti, Ulrika Warpman Berglund, Pål Stenmark, Thomas Helleday

**Affiliations:** 1 Science for Life Laboratory, Department of Oncology-Pathology, Karolinska Institutet, S-171 76 Stockholm, Sweden; 2 Department of Biochemistry and Biophysics, Stockholm University, S-106 91 Stockholm, Sweden; 3 Department of Clinical and Molecular Medicine, Norwegian University of Science and Technology, Trondheim, Norway; 4 Department of Pathology, St. Olavs Hospital, Trondheim, Norway; 5 Sheffield Cancer Centre, Department of Oncology and Metabolism, University of Sheffield, Sheffield S10 2RX, UK

## Abstract

Nucleotides in the free pool are more susceptible to nonenzymatic methylation than those protected in the DNA double helix. Methylated nucleotides like O6-methyl-dGTP can be mutagenic and toxic if incorporated into DNA. Removal of methylated nucleotides from the nucleotide pool may therefore be important to maintain genome integrity. We show that MutT homologue 1 (MTH1) efficiently catalyzes the hydrolysis of O6-methyl-dGTP with a catalytic efficiency similar to that for 8-oxo-dGTP. O6-methyl-dGTP activity is exclusive to MTH1 among human NUDIX proteins and conserved through evolution but not found in bacterial MutT. We present a high resolution crystal structure of human and zebrafish MTH1 in complex with O6-methyl-dGMP. By microinjecting fertilized zebrafish eggs with O6-methyl-dGTP and inhibiting MTH1 we demonstrate that survival is dependent on active MTH1 *in vivo*. O6-methyl-dG levels are higher in DNA extracted from zebrafish embryos microinjected with O6-methyl-dGTP and inhibition of O6-methylguanine-DNA methyl transferase (MGMT) increases the toxicity of O6-methyl-dGTP demonstrating that O6-methyl-dGTP is incorporated into DNA. MTH1 deficiency sensitizes human cells to the alkylating agent Temozolomide, a sensitization that is more pronounced upon MGMT inhibition. These results expand the cellular MTH1 function and suggests MTH1 also is important for removal of methylated nucleotides from the nucleotide pool.

## INTRODUCTION

DNA can be methylated nonenzymatically by environmental methylating agents, chemotherapeutics such as Temozolomide (1) and natural cellular methyl donors like S-adenosylmethionine (SAM) (2). Free nucleotides are reported to be ∼190–13 000 times more susceptible to methylation damage, depending on the site of methylation, compared to when present in the DNA (3). This is likely due to a higher accessibility of free nucleotides compared to when present in the densely packed DNA. The order of susceptibility for methylation of free nucleotides by N-methyl-N-nitrosourea was found to be dependent on the site on the base, with methylations at the N1 position of adenine being the most common, followed by the N3 position of adenine. The most susceptible position on guanine was the N7 position followed by the O6 position (3). If incorporated into DNA these methylated nucleotides can produce mutations and toxic DNA lesions. For example, N3‐methyl-A blocks replication, O6-methyl-G induces G:C to A:T mutations (4) and causes DNA strand breaks and replication fork collapse (5) and N7-methyl-G gives rise to mutagenic apurinic sites and blocks DNA replication (6–8).

The importance of removing methylations from DNA to prevent mutations and DNA lesions is reflected in the presence of an array of DNA-glycosylases that remove methylated bases from the DNA. N3‐methyladenine, N7-methylguanine and N3-methylguanine are removed from DNA by alkyladenine-DNA glycosylase (9,10). AlkB homologue dioxygenases repair alkylated DNA containing N1-methyladenine by oxidative demethylation (11–13). O6-methyl-G adducts are removed from the DNA in a suicide reaction where the methyl group is transferred to a cysteine in the active site of O6-methylguanine-DNA methyltransferase (MGMT) (14). Given the importance of preventing unwanted methylations in the DNA for genome integrity and the high susceptibility to methylation of the free nucleotide pool we argued that removal of methylated dNTPs from the free nucleotide pool also would be important. In addition, the presence of O6-methyl-guanosine in RNA has been shown to alter the fidelity and the translation rate of the ribosome (15). Hence, it would also be of importance to remove O6-methyl-GTP from the NTP pool to decrease cellular toxicity.

MutT homologue 1 (MTH1) belongs to the NUDIX protein family and is known to be responsible for removal of oxidized purine nucleoside triphosphates from the nucleotide pool (16). We set out to investigate if MTH1 also can catalyze the hydrolysis of methylated nucleotides. We here show that MTH1 constitutes another line of defense against DNA methylations by efficiently catalyzing the hydrolysis of O6-methyl-dGTP. We reveal the importance of MTH1 activity in cells exposed to O6-methyl-dGTP for survival *in vivo* and demonstrate that the ability of MutT homologues to hydrolyze O6-methyl-dGTP has been conserved through evolution and is exclusive to MTH1 among human NUDIX enzymes.

## MATERIALS AND METHODS

### Human MTH1 expression and purification

MTH1 expression and purification was performed as previously described (17). Briefly, human MTH1 (hMTH1, NUDT1) was expressed from pET28a(+) (Novagen), containing MTH1 cDNA optimized for expression in *Escherichia coli* (*E. coli)*, in strain BL21 DE3 (Stratagene). His tagged MTH1 was affinity purified using HisTrap HP (GE Healthcare). His tag was removed by thrombin digestion (Novagen) and the MTH1 protein was further purified using anion exchange chromatography at pH 7.5 using a MonoQ column (GE Healthcare). The identity and purity of the protein was confirmed using SDS-PAGE gel analysis and MALDI-TOF mass spectrometry (MS).

### MTH1 activity determination

In order to investigate if MTH1 can hydrolyze methylated dGTPs the activity of MTH1 was tested with the methylated dGTPs that were available for purchasing, O6-methyl-dGTP and N2-methyl-dGTP (TriLink Biotechnologies). Activity of MTH1 (5 nM) was tested in MTH1 reaction buffer (100 mM Tris acetate pH 7.5, 40 mM NaCl, 10 mM Magnesium acetate, 1 mM DTT) with 50 μM O6-methyl-dGTP or N2-methyl-dGTP and for comparison with 50 μM dGTP and 50 μM 8-oxo-dGTP, at 22°C using a reaction time of 30 min. Formed PPi was converted to Pi by using an excess of *E. coli* PPase (0.2 U/ml) (produced by the Protein Science Facility at the Karolinska Institute) as described (18) and Pi was detected by addition of malachite green reagent followed by incubation with shaking for 15 min and measurement of the absorbance at 630 nm (19). The amount of Pi present in the sample was determined by converting *A*_630_ to [Pi] by using a Pi standard curve. Activity differences between samples in quadruplicate were tested for statistical significance using multiple comparisons and one-way ANOVA (Alpha 0.05) using the GraphPad Prism 6.0 software. Activity was also tested with 50 μM O6-methyl-GTP and GTP using 45 nM MTH1 in MTH1 reaction buffer with PPase (0.2 U/ml). Reactions were incubated at 22°C for 30 min and were run in quadruplicate. Malachite green reagent was added to detect formed Pi as described above. Statistic significance was tested using *t*-test (two-tailed *P*-value) using the GraphPad Prism 6.0 software.

The observed activity of MTH1 with O6-methyl-dGTP prompted us to analyse the MTH1 catalyzed hydrolysis of O6-methyl-dGTP more carefully. 5 mM O6-methyl-dGTP was incubated with MTH1 (100 nM) at 22°C in MTH1 reaction buffer pH 8, and the reaction was stopped at different time points by protein denaturation through sample heating at 70°C for 10 min followed by precipitation of denatured protein by centrifugation at 17 000 g for 10 min at 4°C. Supernatants were diluted 1:10 with distilled water and samples were analysed using an Agilent 1100 HPLC system equipped with a Hypercarb column (100 mm × 2.1 mm, Thermo Fisher Scientific), by using a basic pH method. H_2_O (containing 10 mM NH_4_HCO_3_ pH 10) and Acetonitrile were used as mobile phases at a flow rate of 0.5 ml/min. The reaction mixture was separated using a gradient of 0–40% Acetonitrile and a gradient time of 6.0 min and UV light absorbance in the 180−305 nM range was used for detection.

Kinetic parameters of MTH1 with O6-methyl-dGTP and O6-methyl-GTP (TriLink Biotechnologies) were determined by assaying initial rates at substrate concentrations ranging from 0 to 100 μM for O6-methyl-dGTP and 0 to 400 μM for O6-methyl-GTP in MTH1 reaction buffer pH 7.5. Formed PPi was detected using the PPiLight inorganic pyrophosphate assay (Lonza). A PPi standard curve was used to convert assay signal to concentration PPi. The *k*_cat_, *K*_M_ and *k*_cat_/*K*_M_ values were determined by fitting the Michaelis–Menten equation to initial rates monitored in triplicates for each concentration using non-linear regression analysis in the GraphPad Prism Software.

### Docking of N7-methyl-dGMP into MTH1 active site

The most susceptible position of guanine for methylation is the N7 position giving rise to N7-methyl-dGTP in the free nucleotide pool (20). To get an idea of how well N7-methyl-dGTP fits in the active site of MTH1 we performed molecular modelling using Small-Molecule Drug Discovery Suite 2017-1 (Schrödinger, LLC, New York, NY, 2017). The crystal structure of MTH1 in complex with 8-oxo-dGMP (pdb 3ZR0 (17) was prepared using the Protein Preparation Wizard. Briefly, the B chain was deleted and the A chain was processed by automatically assigning bond orders, adding hydrogens, creating zero-order bonds to metals, creating possible disulfide bridges, deleting waters beyond 5.0 Å of het groups, and generating het states at pH 7.0. Protonation and metal charge states were then generated for the het groups and visually inspected, and the most likely states based on H-bonding pattern and state penalty were selected. Two sulfate groups were deleted at this point. The hydrogen bonding network was initially optimized interactively, locking Asp119 and Asp120 as neutral and charged, respectively, and then the remaining species were optimized automatically, by sampling water orientations and optimization of hydroxyls, Asn, Gln and His states using ProtAssign. Subsequently all waters with <4 H-bonds to non-waters were removed, and finally the structure was submitted to a restrained minimization in the OPLS3 force field, until the heavy atom positions had converged to an RMSD of 0.30 Å. Glide docking grids were then generated by centering them onto bound 8-oxo-dGMP. N7-methyl-dGMP was manually created from 8-oxo-dGMP, and low-energy 3D starting conformations of both ligands were then generated using LigPrep. N7-methyl-dGMP was then docked to the active site of MTH1 using Glide XP, without any constraints.

### Production and analysis of methylated dGTP and analysis of MTH1 activity using HPLC

N7-methyl-dGTP could not be purchased presumably due to a short half-life. We therefore produced N7-methyl-dGTP and tested the ability of MTH1 to catalyze the hydrolysis of N7-methyl-dGTP. dGTP was methylated by incubating 50 mM dGTP (Sigma-Aldrich) at 22°C for 20 h with 100 mM MMS diluted in DMSO, or the same volume DMSO, resulting in a dGTP:MMS ratio of 1:2. Methylated dGTP was also generated by incubating 10 mM dGTP at 22°C for 20 h with 300 mM Iodomethane diluted in DMSO-d6:D_2_O ratio 3:1 for analysis using NMR. For assessment of MTH1 activity, MMS treated dGTP and DMSO control were diluted to 5 mM with MTH1 reaction buffer (0.1 M Tris acetate pH 8.0, 40 mM NaCl, 10 mM Magnesium acetate) followed by incubation with 500 nM MTH1 at 22°C. The hydrolysis reaction was stopped at different time points by heat inactivation at 70°C for 10 min followed by centrifugation at 17 000 g for 10 min at 4°C. Reaction mixtures were analysed using HPLC equipped with a Hypercarb column and molecular masses were determined using LC–MS as described below.

### Analysis of nucleotide samples using HPLC–MS and NMR

NMR spectra of dGTP and N7-methyl-dGTP, prepared by methylation of dGTP using MMS and Iodomethane, were recorded on a Bruker DRX-400. Chemical shifts are expressed in parts per million (ppm) and referenced to the residual solvent peak. Molecular masses were determined using HPLC–MS utilizing an Agilent MSD mass spectrometer connected to an Agilent 1100 HPLC system equipped with an XTerra column (MSC18, 50 mm × 3.0 mm) and by using a basic pH method utilizing H_2_O (containing 10 mM NH_4_HCO_3_ pH 10) and Acetonitrile as mobile phases. The HPLC method used a flow rate of 1 ml/min, a gradient of 0–10% Acetonitrile and a gradient time of 3.0 min. For detection absorbance in the 180−305 nM range was used. Masses were determined using electrospray ionization MS.

### Production of human NUDIX enzymes

Expression constructs of NUDT4 (DIPP2α) and NUDT9 were constructed by amplifying cDNA that was synthesized from HL60 cell RNA isolated in house, and was subcloned into pET28a(+) (Novagen). Expression constructs of NUDT2, NUDT7, NUDT17 and NUDT18 were produced by subcloning of cDNA, purchased as codon optimized for *E. coli* expression from GeneArt (Life technologies), into pET28a(+) (Novagen). NUDT21 and NUDT22 cDNAs were purchased from Source BioScience and subcloned into pET28a(+). Correctness of expression constructs sequences was verified by sequencing. Expression constructs of NUDT3 (aa 8–172), NUDT5, the catalytic subunit of NUDT6, NUDT10 (variant AAH50700), NUDT11 (aa 13–164), NUDT12, NUDT14, NUDT15 and NUDT16 (variant AAH31215) all in pNIC28 were kind gifts from SGC Stockholm. All NUDIX proteins were expressed and purified as N-terminally His-tagged proteins apart from NUDT10 and NUDT11 that were C-terminally tagged. Expression strain was *E. coli* BL21(DE3) R3 pRARE2 and expression was performed at 18°C. Proteins were purified by the Protein Science Facility (PSF) at Karolinska Institute, Stockholm. The N-terminally His-tagged NUDIX proteins were purified using HisTrap HP (GE Healthcare) followed by gel filtration on HiLoad 16/60 Superdex 75 (GE Healthcare). NUDT2 was produced in BL21 DE3 (Life Technologies) by overnight expression at 18°C and was purified using HisTrap HP followed by ion exchange chromatography on HP monoQ (GE Healthcare). The NUDIX proteins were concentrated and stored at –80°C in storage buffer (20 mM HEPES pH 7.5, 300 mM NaCl, 10% Glycerol and 0.5 mM TCEP). The purity of protein preparations was examined using SDS-PAGE followed by Commassie staining and the correct mass of the purified proteins was verified using MS.

### O6-methyl-dGTP activity screening of human NUDIX hydrolases

The activity of human NUDIX proteins with 50 μM O6-methyl-dGTP was assessed in reaction buffer (100 mM Tris acetate pH 7.5, 40 mM NaCl, 10 mM Magnesium acetate, 1 mM DTT) at 22°C. Reactions were performed in presence of 0, 5 or 200 nM NUDIX protein diluted in reaction buffer, containing an excess of *E. coli* PPase (0.2 U/ml) or without coupled enzyme. After 30 min incubation with shaking malachite green reagent was added to detect the formed inorganic phosphate (19). After 15 min incubation with shaking 10 μl 0.4 M Sodium citrate was added to the 40 μl reaction mixture to stop the development. Absorbance was measured at 630 nm using an EnVision plate reader (Perkin Elmer).

### Activity assessment of MTH1, NUDT17 and NUDT18 with methylated nucleoside triphosphates

To further investigate the capacity of MTH1 to catalyze the hydrolysis of methylated nucleoside triphosphates we tested the activity of 5 nM MTH1 with a panel of methylated (d)NTPs and for comparison the corresponding canonical (d)NTP, when available, at 50 μM. The nucleotides included in the substrate screen were: dATP (Sigma Aldrich, DNTP-100), ATP (Sigma Aldrich, A26209), N1-methyl-ATP (Jena Biosciences NU-1027), N6-methyl-ATP (Jena Biosciences, NU-1101), UTP (Sigma Aldrich, U1006), 5-methyl-UTP (Jena Biosciences, NU-880), dCTP (Sigma Aldrich, DNTP-100), 5-methyl-dCTP (TriLink Biotechnologies, N-2026), 5-methyl-CTP (Jena Biosciences, NU-1138), GTP (Sigma Aldrich, G3776), 7-methyl-GTP (Sigma Aldrich, M6133), O6-methyl-GTP (TriLink Biotechnologies, N-1031), dGTP (Sigma Aldrich, 27-1870-04) and for comparison O6-methyl-dGTP (TriLink Biotechnologies, N-2027). The reaction buffer was 100 mM Tris acetate pH 8.0, 40 mM NaCl and 10 mM Magnesium acetate and contained 0.2 U/ml PPase. Formed Pi was after 30 min incubation with shaking at 22°C detected using the malachite green assay by measuring the absorbance at 630 nm using a Hidex plate reader. Signal from PPase only controls were subtracted from sample signals. Since NUDT17 and NUDT18 showed some activity with O6-methyl-dGTP we decided to test the activity with these enzymes with the above mentioned panel of nucleotides using the same assay conditions as for MTH1 but using 200 nM NUDT17 and NUDT18. Two independent experiments with samples in triplicate were performed.

### Production of MTH1 proteins from various species

cDNAs for clMTH1 (dog), ssMTH1 (pig), mmMTH1 (mouse), rnMTH1 (rat) and atNUDT1 (*Arabidopsis thaliana* plant) were purchased as codon optimized for *E. coli* expression from GeneArt (Thermo Fisher Scientific) or Eurofins and subcloned into pET28a(+) (Novagen). The zfMTH1 (zebrafish) expression construct has been described previously (21). Proteins were expressed in *E. coli* BL21 (DE3) R3 pRARE2 or BL21 (DE3) T1R pRARE2 (mmNUDT1) in Terrific Broth (TB) media at 18°C for 16 h after induction by 0.5 mM IPTG. Proteins were purified from the bacterial lysate using HisTrap HP followed by gel filtration on HiLoad 16/60 Superdex 75. *E. coli* MutT was produced as described previously (22). Purified proteins were analysed for purity using SDS-PAGE and analysed by MS to verify the correct protein mass. Proteins were stored as aliquots at –80°C in 20 mM HEPES buffer pH 7.5, 500 mM NaCl, 10% Glycerol and 0.5 mM TCEP.

### Determination of activity of MTH1 from different species with O6-methyl-dGTP and 8-oxo-dGTP

Activity of MTH1 from different species was tested by incubating 1.5 nM of MTH1 from respective species with 75 μM 8-oxo-dGTP or 75 μM O6-methyl-dGTP in MTH1 reaction buffer (100 mM Tris acetate pH 8.0, 40 mM NaCl, 10 mM Magnesium acetate, 1 mM DTT) for 15 min. Formed PPi was detected using PPiLight Inorganic Pyrophosphate Assay kit from Lonza by measuring luminescence with time using a Hidex Sense plate reader.

### Production of zfMTH1 for crystallization

The zfMTH1 protein for crystallographic studies was expressed in *E. coli* BL21 (DE3; Novagen) by induction with 0.5 mM IPTG at an OD_600_ of 1.0 in Terrific Broth media and further growth at 18°C overnight. Bacteria were harvested by centrifugation and lysate was prepared by adding Lysis buffer (100 mM HEPES, pH 8.0, 500 mM NaCl, 10% (v/v) Glycerol, 0.5 mM TCEP) containing lysozyme, 5 mM MgSO_4_, DNAse, and protease inhibitor cocktail (Roche) followed by high-pressure homogenization and centrifugation. His-tagged zfMTH1 was purified on gravity flow column (Econo-Pac Chromatography column, Bio-Rad) after incubation with Ni-NTA (1.0 ml/50 ml cleared lysate) and 10 mM Imidazole, washed with Buffer A (20 mM HEPES buffer pH 7.5, 500 mM NaCl, 10% Glycerol, and 0.5 mM TCEP) fortified with 10 mM Imidazole followed by elution of the protein with Buffer A fortified with 500 mM Imidazole. MTH1 containing fractions were pooled and loaded on a PD-10 desalting column (GE Healthcare) equilibrated with Buffer B (20 mM HEPES pH 7.5, 300 mM NaCl, 10% Glycerol, and 0.5 mM TCEP). Eluted protein was cleaved by Thrombin (GE Healthcare) over night at 4°C, then purified using Ni-NTA with Buffer B fortified with 10 mM Imidazole. Fractions containing zfMTH1 were pooled, loaded on a Superdex 75 16/60 column (GE Healthcare), and purified using Buffer B. Fractions containing zfMTH1 were pooled and purity was analysed on SDS-PAGE. Protein was concentrated using Vivaspin 20 (Sartorious Stedim), 10 kDa MWCO. Concentration was determined by measuring *A*_280_ and using a calculated extinction coefficient of 24 980 M^−1^ cm^−1^.

### Crystallization and structure determination

3 mM O6-methyl-dGTP and 2 mM TCEP was added to the human MTH1 protein. Sitting drop vapor diffusion experiments were performed at 20°C, and human MTH1 (16.9 mg/ml in 20 mM Tris–HCl pH 7.4, 150 mM NaCl and 5% (v/v) Glycerol) was mixed with reservoir solution (26% (w/v) PEG6000, 100 mM Sodium Acetate pH 3.7 and 200 mM LiSO_4_) in a 1:1 ratio. Diffraction quality crystals appeared after approximately 3 days, and were flash frozen in liquid nitrogen. Data collection was performed at 100 K at beamline 14.1 at BESSY, Germany. 2.5 mM O6-methyl-dGTP and 6 mM MgCl_2_ were added to the zfMTH1 protein. Crystals were grown using the sitting drop vapor diffusion technique. ZfMTH1 (45.17 mg/ml) was mixed with reservoir solution (200 mM CaCl_2_, 100 mM MES pH 6.0, 20% (w/v) PEG6000) in a 1:3 ratio. Diffraction quality crystals appeared after about 3 days at 20°C. Crystals were cryo-protected by soaking in 50% mother liquor supplemented with 42.5% Glycerol, and were subsequently flash frozen in liquid nitrogen. Data collection was performed at 100 K at beamline I04-1 at Diamond, United Kingdom.

Data reduction and processing was carried out using XDS (23) and programs from the CCP4 suite (24). Relevant statistics can be found in Supplementary Table S1. The structures were solved via molecular replacement using Phaser (25), using the previously solved *apo* MTH1 structure (pdb 3ZR1) as search model for the human MTH1 structure and the previously solved zfMTH1 structure (pdb 5HZX) as search model for the zfMTH1 structure. A few cycles of refinement in Refmac5 (24,26), interspersed with manual building in Coot (27,28), were needed to complete the models. Water molecules were automatically placed in the maps, using a *F*_O_ – *F*_C_ Fourier difference map cutoff of 3σ, and were subsequently validated to ensure correct positioning. All structure figures were prepared using PyMOL (Schrödinger). Ramachandran statistics were generated using MolProbity (29,30). The structures have been deposited in the protein data bank with accession codes 5OTM (hMTH1) and 5OTN (zfMTH1).

### Structure comparisons

RMSD comparisons between structures for Cα-atoms were obtained using the protein structure comparison service PDBeFold at European Bioinformatics Institute (http://www.ebi.ac.uk/msd-srv/ssm), authored by Krissinel and Henrick (31).

### Determination of activity with O6-methyl-dGTP and dGTP of human and zebrafish MTH1

To assess the relative activity of zfMTH1 with O6-methyl-dGTP versus dGTP and to compare the activity of zfMTH1 with the activity of human MTH1, 5 nM of the respective enzymes were incubated with 100 μM dGTP or O6-methyl-dGTP in MTH1 reaction buffer (100 mM Tris acetate pH 8.0, 40 mM NaCl, 10 mM Magnesium acetate, 1 mM DTT) for 20 min. Formed PPi was converted to Pi by using an excess of *E. coli* PPase followed by detection of Pi using malachite green reagent and absorbance measurement at 630 nm. A Pi standard curve was used to convert absorbance to formed Pi.

### Inhibition of zfMTH1

TH588 and TH1579 were previously identified as potent inhibitors to human MTH1 and prepared as previously described (22,32). To determine the potency of these compounds towards zfMTH1, TH588 and TH1579 were serially diluted in 1:3 dilution series resulting in 12 final inhibitor concentrations ranging from 10 μM to 0.06 nM. Each inhibitor concentration was run in duplicate. Reaction buffer was 100 mM Tris acetate pH 8.0, 40 mM NaCl, 10 mM Magnesium acetate, 1 mM DTT and 0.005% Tween 20. Final concentration of zebrafish MTH1 (zfMTH1) and dGTP were 5 nM and 100 μM, respectively. *E. coli* PPase was added to a final concentration of 0.2 U/ml. Control reactions, lacking inhibitor or enzyme, were included on the assay plate. The reactions were incubated with shaking at 22°C for 20 min followed by addition of malachite green assay reagent (19) and incubation with shaking for 15 min at 22°C. Absorbance was measured at 630 nm using a Hidex Sense plate reader. IC_50_-values were determined by fitting the equation: log[inhibitor] versus response – variable slope to the inhibition data using nonlinear regression in the GraphPad Prism Software.

### Zebrafish survival experiment with O6-methyl-dGTP and MTH1 inhibitors

Zebrafish (TL strain) were raised and staged according to standard protocols. Fertilized eggs were injected with ∼2 nl of 0.15 mM O6-methyl-dGTP (TriLink Biotechnology) in injection buffer (9 μM spermine, 0.21 μM spermidine, 0.3% phenol red), distributed to six-well plates with 3 ml E3 medium (5 mM NaCl, 0.17 mM KCl, 0.33 mM CaCl_2_×2 H_2_O, 0.33 mM MgSO_4_) and exposed to 1.5 μM TH588, 1.5 μM TH1579 or DMSO added to the water within 15 min after injection. Twenty four hours after injection, embryo viability was analysed (severely malformed individuals or embryos lacking heart beat were counted as dead).

### Survival and DNA analysis of O6-methyl-dGTP, MTH1 and MGMT inhibitor treated zebrafish

An enhancement of O6-methyl-dGTP toxicity by MGMT inhibition would suggest that O6-methyl-dGTP toxicity is exerted through incorporation into DNA. To test this O6-methyl-dGTP was injected into fertilized zebrafish eggs as described above, MGMT inhibitor Lomeguatrib (1 μM) (selleckchem.com), TH588 (1.5 μM) or DMSO were added to the water, either alone or in combination (40–60 eggs in each group). Viability of zebrafish embryos was assessed after 24 h. In addition, DNA from the different treatment groups was isolated using the DNeasy Blood & Tissue Kit (Qiagen) and analysed for presence of O6-methyl-dG and N7-methyl-dG using LC–MS/MS. Prior to nucleoside analysis, RNA in the DNA isolates was degraded by incubating samples with 10 μg RNase (Sigma-Aldrich) in 10 mM Ammonium bicarbonate pH 7.0, 1 mM MgCl_2_, and 0.1 mM Deferoxamine mesylate (DFO; Santa Cruz Biotechnologies) at 37°C for 30 min. Free nucleosides and nucleotides were then removed from samples by centrifugation through 30 kDa molecular weight cut-off columns (Merck) and re-dissolved in UHPLC-grade water. After RNAse pre-treatment, 15 μg DNA were hydrolyzed in 50 μl buffer (10 mM Ammonium acetate pH 5.5, 1 mM MgCl_2_, and 0.1 mM ZnCl_2_) containing 0.8 U nuclease P1 from *P. citrinum* (Sigma-Aldrich), 80 U Benzonase^®^ nuclease (Sigma-Aldrich), and 0.2 U Alkaline phosphatase from *E. coli* (Sigma-Aldrich) at 37°C for 1 h. The reactions were stopped by cooling on ice, and proteins were precipitated by adding three volumes ice-cold Acetonitrile and centrifuging at 16 000 g for 30 min. Supernatants were then lyophilized at –80°C to dryness. Finally, the samples were re-dissolved in 30 μl water for LC/MS/MS analysis, of which 5 μl were diluted 5000-fold to measure the four canonical nucleosides and 20 μl were used to measure modified nucleosides. Modified nucleosides were analysed with an Agilent 6495 triple quadrupole LC/MS/MS system with an Agilent EclipsePlusC18 RRHD column (2.1 × 150 mm, 1.8 μm particle size). The mobile phases were (A) UHPLC-grade water and (B) UHPLC-grade methanol, both containing 0.1% UHPLC-grade formic acid. The HPLC method used a flow rate of 300 μl/min with 5% B to 2.5 min, ramp to 13% B at 3 min, ramp to 17.16% B at 5.5 min, hold at 35% B from 5.5 to 7 min, ramp to 5% at 8 min, and equilibration with 5% B from 7 to 11.5 min. Unmodified nucleosides were measured on an API5000 triple quadrupole mass spectrometer (Applied Biosystems) with an Acentis^®^ Express C18 column (0.5 × 150 mm, 2.7 μm particle size). The HPLC method used a flow rate of 150 μl/min with an isocratic flow of 25% B for 3 min with the column heated to 40°C. The mass transitions used were 282.1 → 166.1, 252.1 → 136, 228.1 → 111.9, 268.1 → 152, and 243.1 → 127 m/z for met-dG, dA, dC, dG, and dT, respectively.

### Activity test of zfMTH1 with Ca^2+^ and Mg^2+^

In order to test if Mg^2+^ can be replaced by Ca^2+^ with retained zfMTH1 activity the activity of zfMTH1 with 100 μM dGTP was tested at 0, 0.125, 0.25, 0.5 and 1 nM zfMTH1 in reaction buffer (100 mM Tris acetate pH 8.0, 40 mM NaCl, 1 mM DTT) supplemented with 10 mM MgCl_2_ or CaCl_2_. After 20 min, the reaction was stopped by heat inactivation at 90°C for 10 min. After cooling to room temperature formed PPi was detected using PPiLight inorganic pyrophosphate assay (Lonza) according to the manufacturers’ recommendations.

### Analysis of role of MTH1 for apoptosis and viability in cells treated with Temozolomide

To analyse the effect of MTH1 on the sensitivity of cells to the alkylating agent Temozolomide, which exerts its toxicity primarily through methylation of O6-guanine, we made use of two glioblastoma cell lines, U251 and U251-MTH1 in which the MTH1 gene had been deleted using CRISPR/Cas9. These two cell lines were kind gifts from Dr Massimo Squatrito. MTH1 knockout was verified using western blotting using rabbit anti-MTH1 (Novus Biologicals). Cells were seeded in MEM medium (Gibco), 10% FBS (Gibco), 50 μg/ml Pen/Strep (Gibco), 1% MEM Non-Essential Amino Acids Solution (100×) (Gibco) and 1 mM Sodium pyruvate (Gibco) and treated with DMSO, Temozolomide (15 μM, Sigma-Aldrich) or Lomeguatrib (20 μM, selleckchem.com) or with both Temozolomide (15 μM) and Lomeguatrib (20 μM). Caspase 3/7 activity was monitored as a measure of early apoptosis after 48 h of treatment (800 cells were seeded in each well of a 384-well plate) at eight different Temozolomide and Lomeguatrib concentrations ranging from 0 to 100 μM, using the Caspase-Glo 3/7 assay (Promega) according to the manufacturer's recommendations. For assessment of cell viability, 400 cells were seeded in each well of a 384-well plate and Temozolomide and Lomeguatrib were added as described for the apoptosis assay. Cell viability was measured after 96 h using Resazurin as previously described (22).

## RESULTS

### MTH1 efficiently catalyzes O6-methyl-dGTP hydrolysis

**Figure 1.**
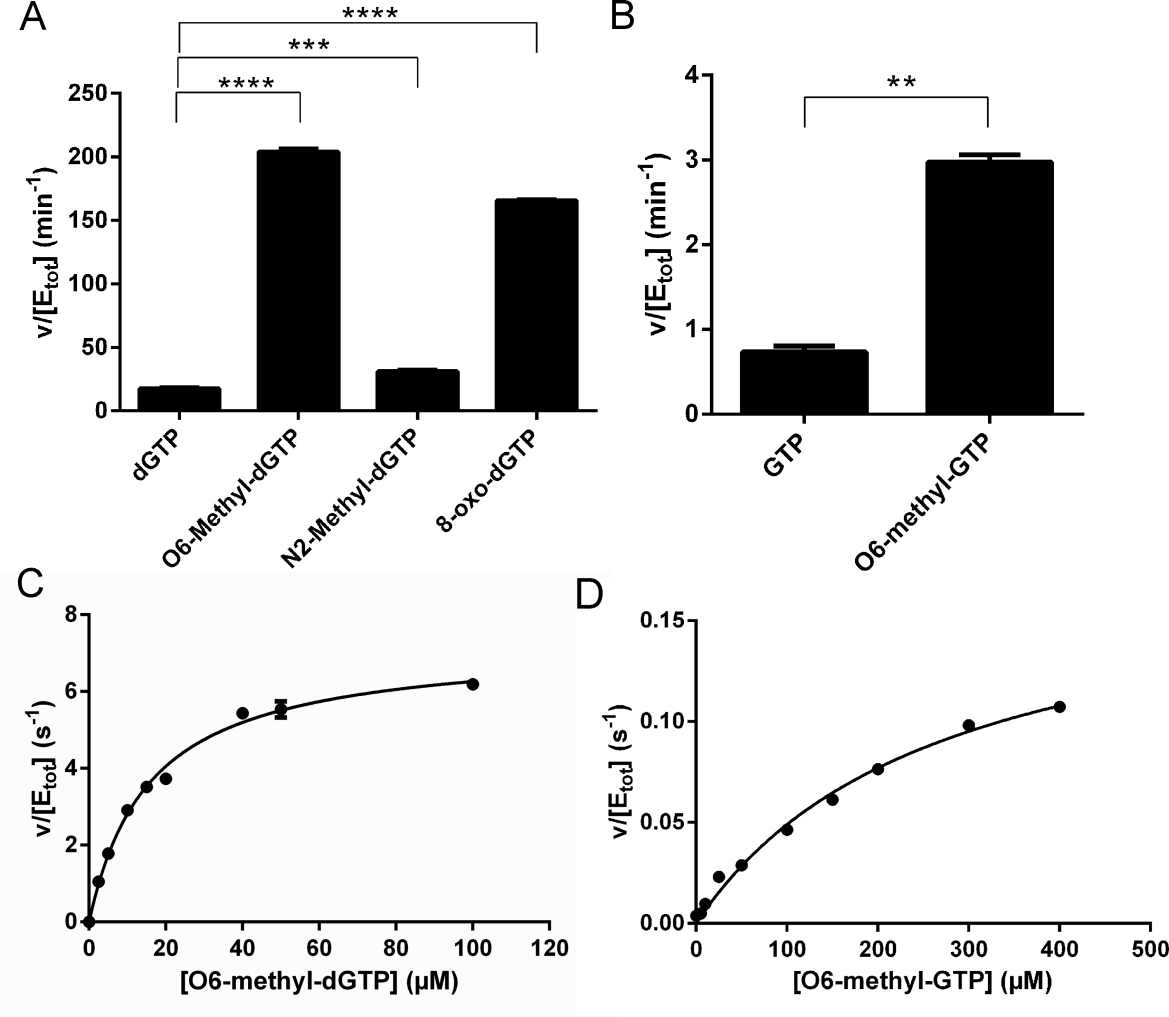
MTH1 is an efficient catalyst of O6-methyl-dGTP hydrolysis. (**A**) Activity assessment of MTH1 with O6-methyl-dGTP and N2-methyl-dGTP in comparison to dGTP and 8-oxo-dGTP. Activity of 5 nM MTH1 was tested with 50 μM substrate in MTH1 reaction buffer with PPase (0.2 U/ml). Formed Pi was detected using the malachite green reagent. Activity differences between samples in quadruplicate were found to be statistically significant by multiple comparisons using One way Anova in the GraphPad Prism 6.0 software. (**B**) Activity of MTH1 (45 nM) with 50 μM O6-methyl-GTP and GTP was measured as in (A) with samples in quadruplicate. Statistic significance was analysed using Paired Two-tailed T-test using the GraphPad Prism 6.0 software. (**C**) Saturation curve for MTH1 with O6-methyl-dGTP were produced by determining initial rates using 1.25 nM MTH1 and O6-methyl-dGTP ranging in concentration between 0 and 40 μM. (**D**) Saturation curve for MTH1 with O6-methyl-GTP. 50 nM MTH1 and O6-methyl-GTP ranging from 0 to 400 μM were used. Shown are representative saturation curves out of two independent experiments for each substrate with data points recorded in duplicate. *P* ≤ 0.05 are considered to be statistically significant and are indicated by *, *P* ≤ 0.01 are indicated by **, *P* ≤ 0.001 are indicated by *** and *P* ≤ 0.0001 are indicated by ****.

As methylation damage preferentially affects the dNTP pool (3) and since removal of methylated dNTPs may very well be important for cell survival we tested the ability of MTH1 to sanitize the damaged dNTP pool by catalysing the hydrolysis of methylated nucleotides. Comparison of activities of MTH1 with O6-methyl-dGTP, N2-methyl-dGTP, dGTP and the previously best known MTH1 substrate 8-oxo-dGTP showed that O6-methyl-dGTP was hydrolyzed at a level similar to that of 8-oxo-dGTP (Figure 1A). However, the activity of MTH1 with N2-methyl-dGTP was only slightly higher than with dGTP. We also tested the activity of MTH1 with the corresponding ribonucleotide O6-methyl-GTP and compared it to the activity with GTP (Figure 1B) and found the activity of MTH1 with O6-methyl-GTP to be approximately 4-fold higher than for GTP. However, the activity with O6-methyl-GTP under the used conditions was approximately 70-fold lower compared to with O6-methyl-dGTP. The MTH1 catalyzed hydrolysis of O6-methyl-dGTP was also analysed using HPLC and LC–MS showing that the product formed is O6-methyl-dGMP as expected (Supplementary Figure S1A). For more detailed analysis of the activity of MTH1 with O6-methyl-dGTP and O6-methyl-GTP saturation curves were produced (Figure 1C and D) and kinetic parameters for MTH1 with O6-methyl-dGTP and O6-methyl-GTP were determined (Table 1). Comparison of kinetic parameters for MTH1 with O6-methyl-dGTP with those determined previously for 8-oxo-dGTP under the same conditions shows that *k*_cat_ and *K*_m_ values are similar resulting in *k*_cat_/*K*_m_-values in the same range for these two substrates (Table 1).

**Table 1.** Kinetic parameters of MTH1

	*K*_m_ (μM)	*k*_cat_ (s^−1^)	*k*_cat_/*K*_m_ (M^−1^s^−1^)	
**O6-methyl-dGTP**	15.6 ± 0.2	8.2 ± 0.9	515 000 ± 80 000	n = 2
**O6-methyl-GTP**	236 ± 44	0.3 ± 0.1	1160 ± 580	n = 2
8-oxo-dGTP*	13.2	3.3	246 200	
	10.2	8.6	840 200	

*Data from (17), and (41), *n* denotes number of experiments. *k*_cat_, *K*_M_ and *k*_cat_/*K*_M_ values were determined by fitting the Michaelis–Menten equation to initial rates using non-linear regression analysis utilizing the GraphPad Prism Software. Values shown are average and standard deviations.

### Activity with O6-methyl-dGTP is exclusive to MTH1 among human NUDIX proteins

**Figure 2.**
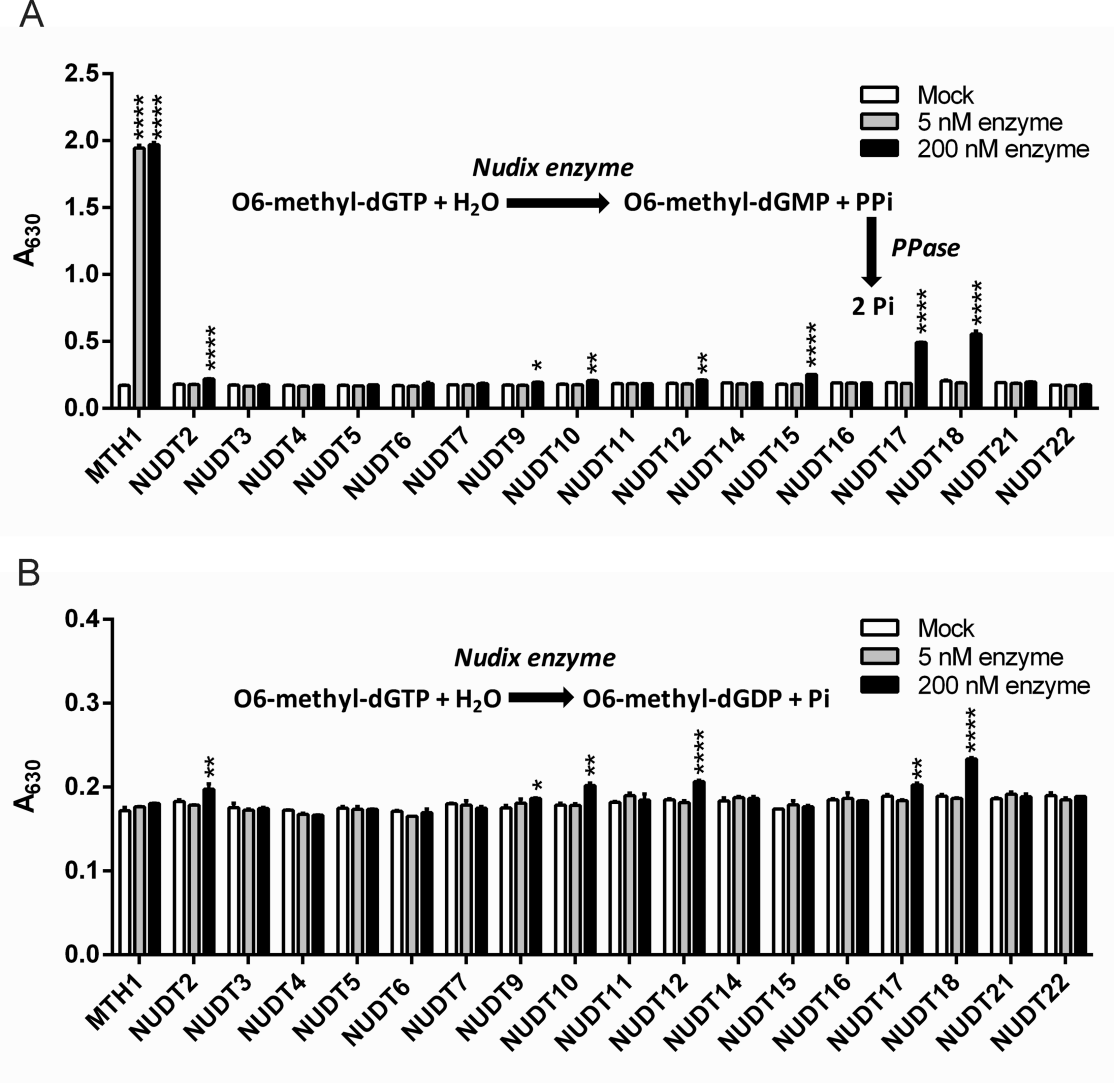
Hydrolysis activity of MTH1 with O6-methyl-dGTP is exclusive among NUDIX hydrolases. Activity screen of human NUDIX proteins with 50 μM O6-methyl-dGTP was tested using 0, 5, or 200 nM NUDIX enzyme in presence of an excess of PPase (**A**) monitoring formation of Pi and PPi, or without coupled enzyme (**B**) detecting formation of Pi. Pi was detected using malachite green reagent and measurement of absorbance at 630 nm. Data points were recorded in triplicate. Statistically significant differences in activity compared to the mock control was assessed using multiple comparison and two-way ANOVA in GraphPad Prism 6.0. *P* ≤ 0.05 are considered to be statistically significant and are indicated by *, *P* ≤ 0.01 are indicated by **, *P* ≤ 0.001 are indicated by *** and *P* ≤ 0.0001 are indicated by ****.

In order to examine if MTH1 is the only human NUDIX protein that can catalyze the hydrolysis of O6-methyl-dGTP we produced and tested 17 of the human NUDIX proteins for activity with O6-methyl-dGTP at a low (5 nM) and a high concentration (200 nM) to allow detection also of low activities as previously described with other substrates (33). The reactions were coupled to *E. coli* PPase producing Pi from formed PPi (Figure 2A) or performed without coupled enzyme showing direct formation of Pi (Figure 2B). At 5 nM enzyme only MTH1 hydrolyzed O6-methyl-dGTP to any detectable level giving rise to O6-methyl-dGMP and PPi. Some hydrolysis of O6-methyl-dGTP was also observed by NUDT17 and NUDT18 but only when using 200 nM enzyme in the assay (Figure 2A). Low activity was also observed using 200 nM NUDT2, NUDT9, NUDT10, NUDT12 and NUDT15. 200 nM NUDT18 showed weak hydrolysis of O6-methyl-dGTP to O6-methyl-dGDP and Pi (Figure 2B). Slight production of Pi was also observed for 200 nM NUDT2, NUDT9, NUDT10, NUDT12 and NUDT17 (Figure 2B). To conclude, this experiment clearly shows that MTH1 by far is the best catalyst of O6-methyl-dGTP hydrolysis among human NUDIX proteins.

### Activity screen of MTH1, NUDT17 and NUDT18 with a panel of methylated nucleotides

**Figure 3.**
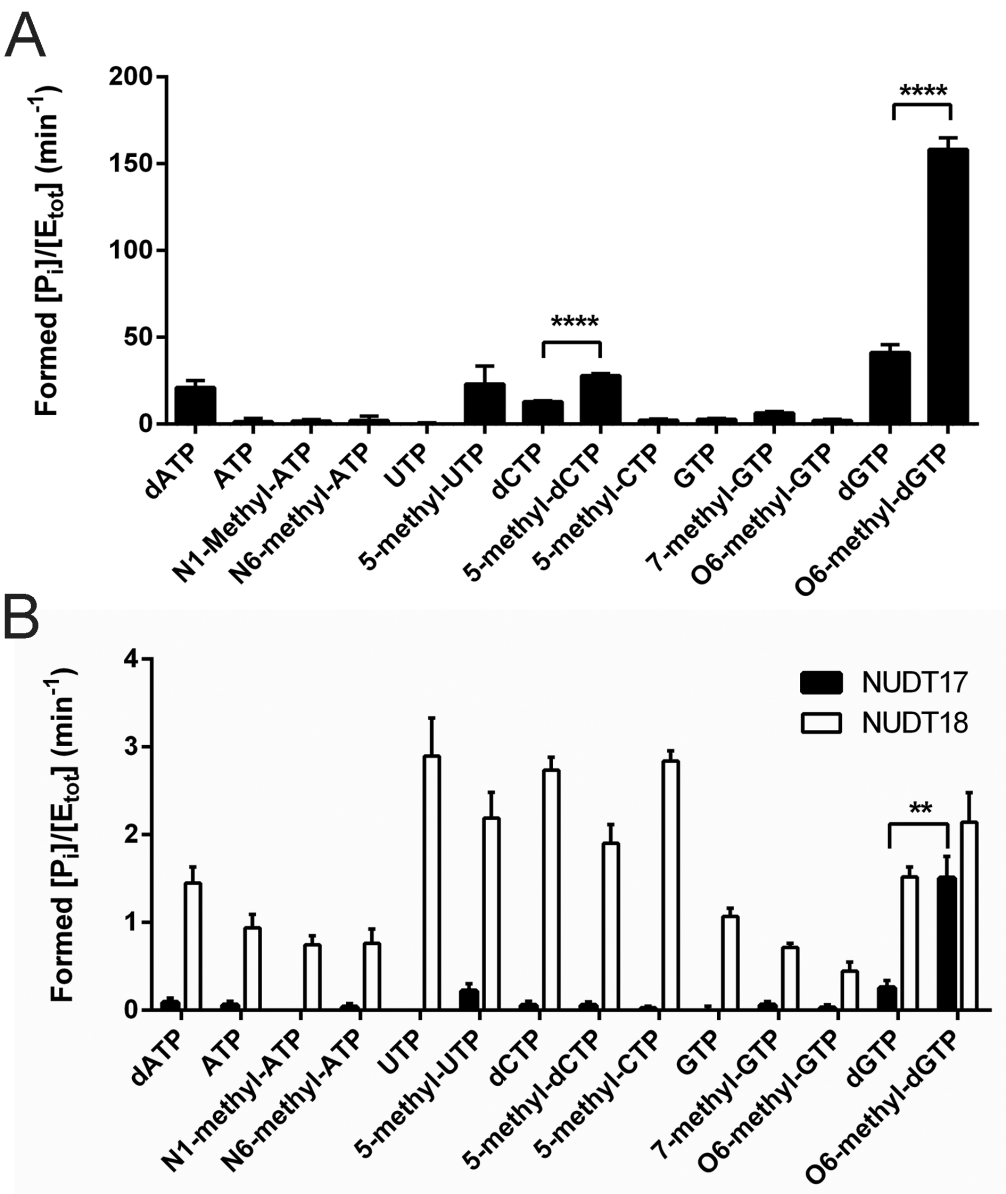
Activity of MTH1, NUDT17 and NUDT18 with methylated and nonmethylated nucleotide. (**A**) MTH1 (5 nM) and (**B**) NUDT17 (200 nM) and NUDT18 (200 nM) were screened for activity with a panel of methylated and nonmethylated deoxyribonucleotides and ribonucleotides by incubation with 50 μM nucleotide at 22°C in MTH1 reaction buffer for 30 min. Formed Pi was detected using the Malachite green assay. Graphs show average and SEM of [Pi] (μM) per min per enzyme concentration ([E_tot_]) (μM) from two independent experiments with data points in triplicate. Statistically significant differences between activity with nonmethylated and methylated nucleotides were determined using two-way ANOVA.

As MTH1 showed considerable activity with O6-methyl-dGTP we sought to investigate if we could identify other methylated (d)NTP species as substrates for MTH1. Analysis of the activity of MTH1 with a panel of methylated (d)NTPs and their canonical counterparts did not identify any of the tested methylated nucleotides as good substrates of MTH1. However, MTH1 did catalyze the hydrolysis of 5-methyl-dCTP more efficiently than dCTP (Figure 3A) although not to the same extent as hydrolysis of dGTP or O6-methyl-dGTP. Since NUDT17 and NUDT18 were found to display some activity with O6-methyl-dGTP (Figure 2A) we also screened these enzymes for activity with the same panel of methylated (d)NTPs, including O6-methyl-GTP, and nonmethylated nucleotides (Figure 3B). Neither NUDT17 nor NUDT18 show any notable activity with any of the tested nucleotides. However, NUDT17 seems to, similar to MTH1, display a preference for methylated nucleotides and catalyzes the hydrolysis of O6-methyl-dGTP and 5-methyl-UTP more efficiently than their canonical counterparts although the displayed activity is very low (Figure 3B). Such preference for methylated nucleotides was not observed for NUDT18.

### N7-methyl-dGTP is not a substrate for MTH1

A second major methylation product of dGTP in addition to O6-methyl-dGTP is N7-methyl-dGTP (20). We therefore decided to investigate if N7-methyl-dGTP could act a substrate for MTH1. We made use of *in silico* docking of N7-methyl-dGMP into the active site of the crystal structure of MTH1 (pdb 3ZR0) (Supplementary Figure S2) to get an idea of how well N7-methyl-dGTP fits in the active site of MTH1. The *in silico* docking produced a low docking score (Glide XP score -14.2 kcal/mol) indicating stronger binding of N7-methyl-dGMP to MTH1 in comparison to 8-oxo-dGMP (–11.7 kcal/mol). This suggests that N7-methyl-dGTP could act as a substrate for MTH1 and MTH1 might protect against the formation of mutagenic apurinic (AP) sites and DNA replication blocks caused by the presence of N7-methyl-dG in the DNA (34) (6). The low docking score is likely to be caused by the positive charge on N7 produced through N7-methylation allowing pi-cation interactions with the aryl rings of W117 and F72 (35).

Encouraged by the result of the docking experiment we set out to produce N7-methyl-dGTP, to be able to test it as an MTH1 substrate, since it is not available for purchasing. Methylations on nucleotides in DNA by MMS mostly result in N7-methylguanine (83%) (36) and only produce minor amounts of O6-methylguanine (0.3% of dN methylations). Based on this information and the fact that N7-methyl-dGTP has been described to be the major product formed after MMS or iodomethane treatment of dGTP (37,38), we successfully generated N7-methyl-dGTP using this route. The formation of N7-methylated dGTP from iodomethane methylation of dGTP was verified by NMR disclosing the presence of a large H8-proton shift arising when the methylation occurs at the N7 position (39,40) (Supplementary Figure S3A and B). By combining HPLC, NMR and LC–MS analysis we could conclude that N7-methyl-dGTP indeed is the major product formed upon MMS treatment of dGTP. Prolonged incubation with excess of methylating reagent resulted in increased formation of a di-methylated product (presumably dGTP with methylations on both N3 and N7) with an elution time of approximately 4.8 min. Addition of MTH1 to the N7-methyl-dGTP preparation (produced by MMS treatment of dGTP) showed no observable hydrolysis of N7-methyl-dGTP as analysed using HPLC even after allowing the reaction to proceed for 4 h with an excess of MTH1. Instead, nonmethylated dGTP in the reaction mixture was hydrolyzed to dGMP under the conditions used (Supplementary Figure S1B). The discrepancy between the results of the *in silico* docking suggesting N7-methyl-dGTP would be a possible MTH1 substrate based on a good fit and experimental data showing it is not emphasizes the importance of performing biochemical experiments to support the results of *in silico* experiments. Altogether, the results strongly suggests that N7-methyl-dGTP is not a substrate of MTH1.

### Activity of MTH1 with O6-methyl-dGTP has been conserved through evolution

**Figure 4.**
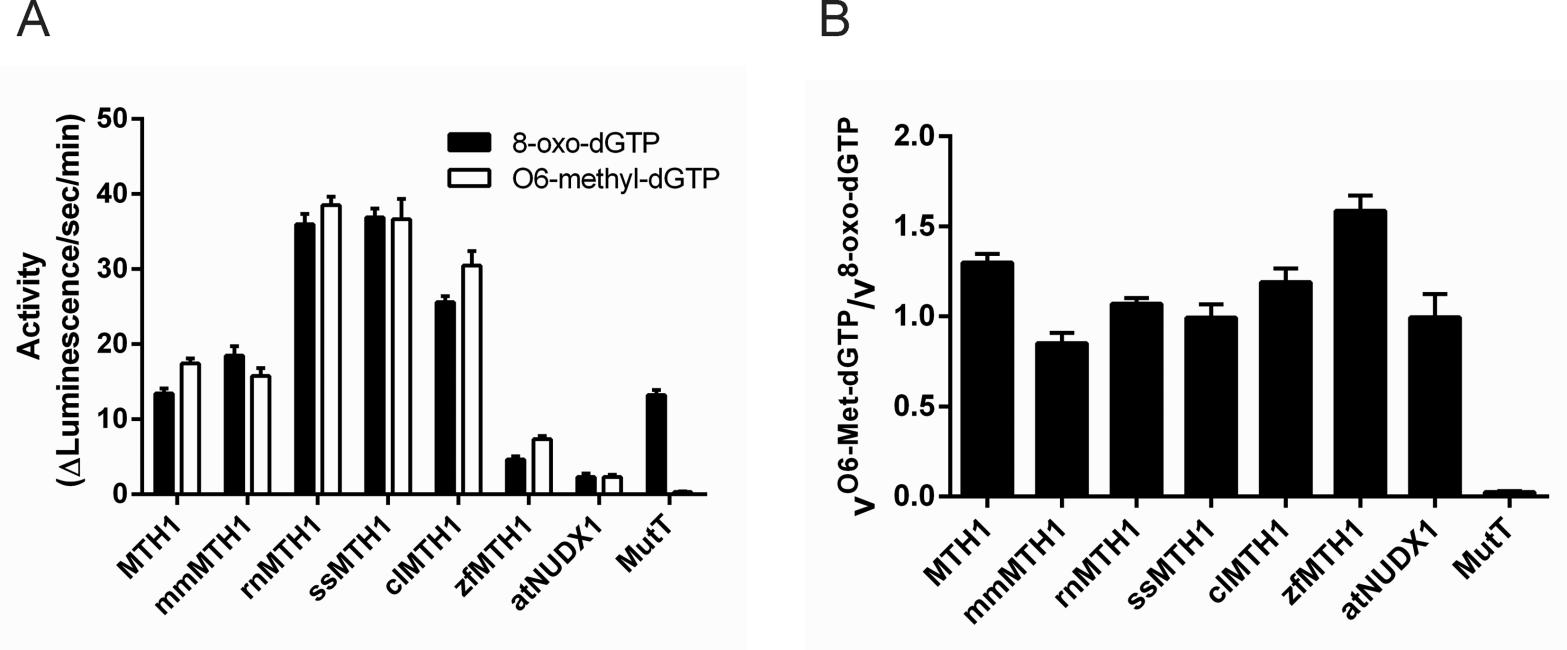
Activity of MTH1 with O6-methyl-dGTP has been conserved through evolution. (**A**) Comparison of activities of MTH1 (NUDT1) (1.5 nM) from different species with 75 μM 8-oxo-dGTP and 75 μM O6-methyl-dGTP (hsNUDT1, human NUDT1; mmNUDT1, mouse NUDT1; rnNUDT1, rat NUDT1; ssNUDT1, pig NUDT1; clNUDT1, dog NUDT1; atNUDX1, Arabidopsis thaliana NUDT1; zfNUDT1, zebrafish MTH1; MutT, *E. coli* MutT). Reaction was performed in MTH1 reaction buffer and reaction time was 15 min. Formed PPi was detected using PPiLight Inorganic Pyrophosphate Assay kit from Lonza. Data points were recorded in triplicate. (**B**) Ratio between activities with O6-methyl-dGTP and 8-oxo-dGTP for MTH1 from different species.

To find out if the high activity of MTH1 with O6-methyl-dGTP is found also in other species and has been conserved through evolution we produced MTH1 from mouse, rat, pig, dog, zebrafish, the plant *Arabidopsis thaliana* and *E. coli* MutT and tested the activity with O6-methyl-dGTP and with 8-oxo-dGTP in parallel. Interestingly, MTH1 homologues from all tested species but *E. coli* were found to catalyze the hydrolysis of O6-methyl-dGTP (Figure 4A). Comparison of activities towards O6-methyl-dGTP and 8-oxo-dGTP show that the activities are similar or even higher towards O6-methyl-dGTP for some of the tested species homologues under the conditions used (Figure 4B). The ability of all tested MTH1 proteins to hydrolyze O6-methyl-dGTP, apart from MutT, suggests that this activity has been conserved through evolution but has arisen after the bacterial, archaeal and eukaryotic branches were separated from each other in the phylogenetic tree of evolution.

### Crystal structures of human and zebrafish MTH1 with bound O6-methyl-dGMP

**Figure 5.**
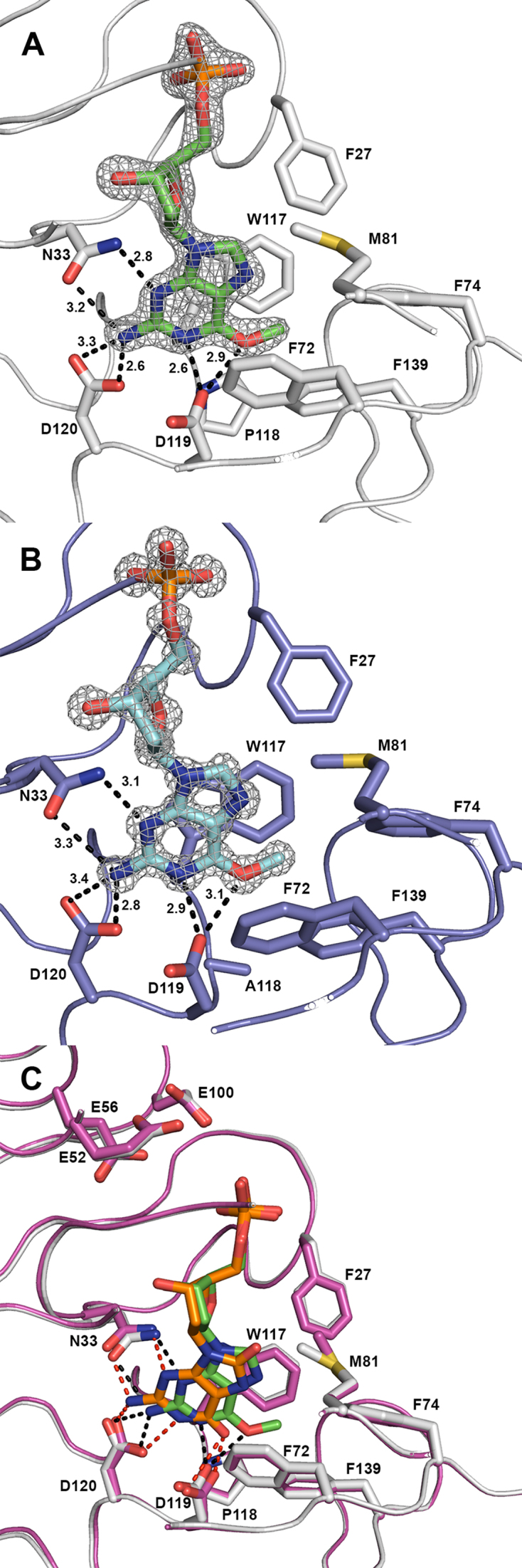
Crystal structures of hMTH1 and zfMTH1 in complex with O6-methyl-dGMP. (**A**) Electron density of O6-methyl-dGMP bound to human MTH1: 2Fo-Fc map at 2.0 σ. hMTH1 is shown in off-white and O6-methyl-dGMP in green. Important binding residues and residues of the hydrophobic pocket are shown as sticks and labelled. Hydrogen bonding distances in Angstroms are shown. (**B**) Electron density of O6-methyl-dGMP bound to zebrafish MTH1: 2Fo-Fc map at 2.0 σ. zfMTH1 is shown in purple and O6-methyl-dGMP in cyan. Important binding residues and residues of the hydrophobic pocket are shown as sticks and are labelled and hydrogen bonding distances in Angstroms are indicated. (**C**) Comparison of O6-methyl-dGMP and 8-oxo-dGMP bound to human MTH1. Human MTH1 is shown in off-white for the O6-methyl-dGMP bound structure and in pink for the 8-oxo-dGMP bound structure. O6-methyl-dGMP is shown in green and 8-oxo-dGMP is shown in orange. Important binding residues and residues of the hydrophobic pocket are shown as sticks and labelled. The E52, E56 and E100 binding the magnesium critical for the catalytic activity are also labelled and shown as sticks.

In order to study the binding mode of O6-methyl-dGTP and explain the high activity of MTH1 with O6-methyl-dGTP co-crystals of both human and zebrafish MTH1 with O6-methyl-dGTP were produced. The structure of human MTH1 with O6-methyl-dGMP, the hydrolysis product, was solved at a resolution of 1.80 Å, while the structure of zfMTH1 with O6-methyl-dGMP was solved at 0.99 Å resolution (see Supplementary Table S1 for details). The binding and electron density for O6-methyl-dGMP in human MTH1 and zfMTH1 is shown in Figure 5A and B, respectively. Within 6 Å of the methylated position, there are a large number of hydrophobic residues, namely F27, F72, F74, M81, W117, P118 (human) or A118 (zebrafish) and F139. When comparing the structure of human MTH1 with O6-methyl-dGMP bound with the previously solved structure of human MTH1 with 8-oxo-dGMP bound, there is a 1.1 Å shift in the position of the guanine base (see Figure 5C), which brings the methylated part of the guanine closer to the hydrophobic pocket. The overall structures of human MTH1 with O6-methyl-dGMP bound and with 8-oxo-dGMP bound (pdb 3ZR0) are virtually identical. The RMSD value for backbone Cα-atoms comparing the two structures, using PDBeFOLD (31) is as low as 0.20 Å showing that the protein backbone does not change when having these two different ligands in the active site. The zfMTH1 structure with O6-methyl-dGMP bound has an RMSD for Cα-atoms of 0.60 Å and 0.74 Å when compared to human MTH1 with O6-methyl-dGMP bound and human MTH1 with 8-oxo-dGMP bound (pdb 3ZR0), respectively. When compared to the previously solved structure of zfMTH1 with inhibitor TH588 bound (pdb 5HZX) the RMSD for zfMTH1 with O6-methyl-dGMP is 0.65 Å. The methylated base O6-methyl-dGMP binds highly similar to both human MTH1 and zfMTH1 as seen in Supplementary Figure S4B. The crystallization condition for zfMTH1 included calcium ions and three calcium ions are found in the structure close to O6-methyl-dGMP and the NUDIX-box motif. The binding interface makes up a crystal contact, with D62 and D92 from a second monomer in the crystal interacting with the calcium ions (see Supplementary Figure S7 for detailed binding). Magnesium ions were found in the active site when solving the structure of human NUDT15 (41). When comparing the binding of calcium to zfMTH1 with the binding of magnesium to human NUDT15 (MTH2, pdb 5BON) in Supplementary Figure S8, two positions overlap but the third calcium ion is not binding anywhere similar to the two remaining magnesium ions to NUDT15. Since zfMTH1 was crystallized in presence of high concentrations of Ca^2+^ it is likely that the presence of Ca^2+^ instead of Mg^2+^ in the crystal structure is due to the used crystallization conditions. However, to exclude that Ca^2+^ can replace Mg^2+^ in the active site in the active enzyme, activity with dGTP was tested in presence of either 10 mM Ca^2+^ or 10 mM Mg^2+^. No activity could be observed in presence of Ca^2+^ (Supplementary Figure S9). To conclude, O6-methyl-dGMP fits very well into the active site of both human and zebrafish MTH1, thus explaining the high activity of MTH1 towards O6-methyl-dGTP.

### Active MTH1 is needed for survival of zebrafish embryos exposed to O6-methyl-dGTP

**Figure 6.**
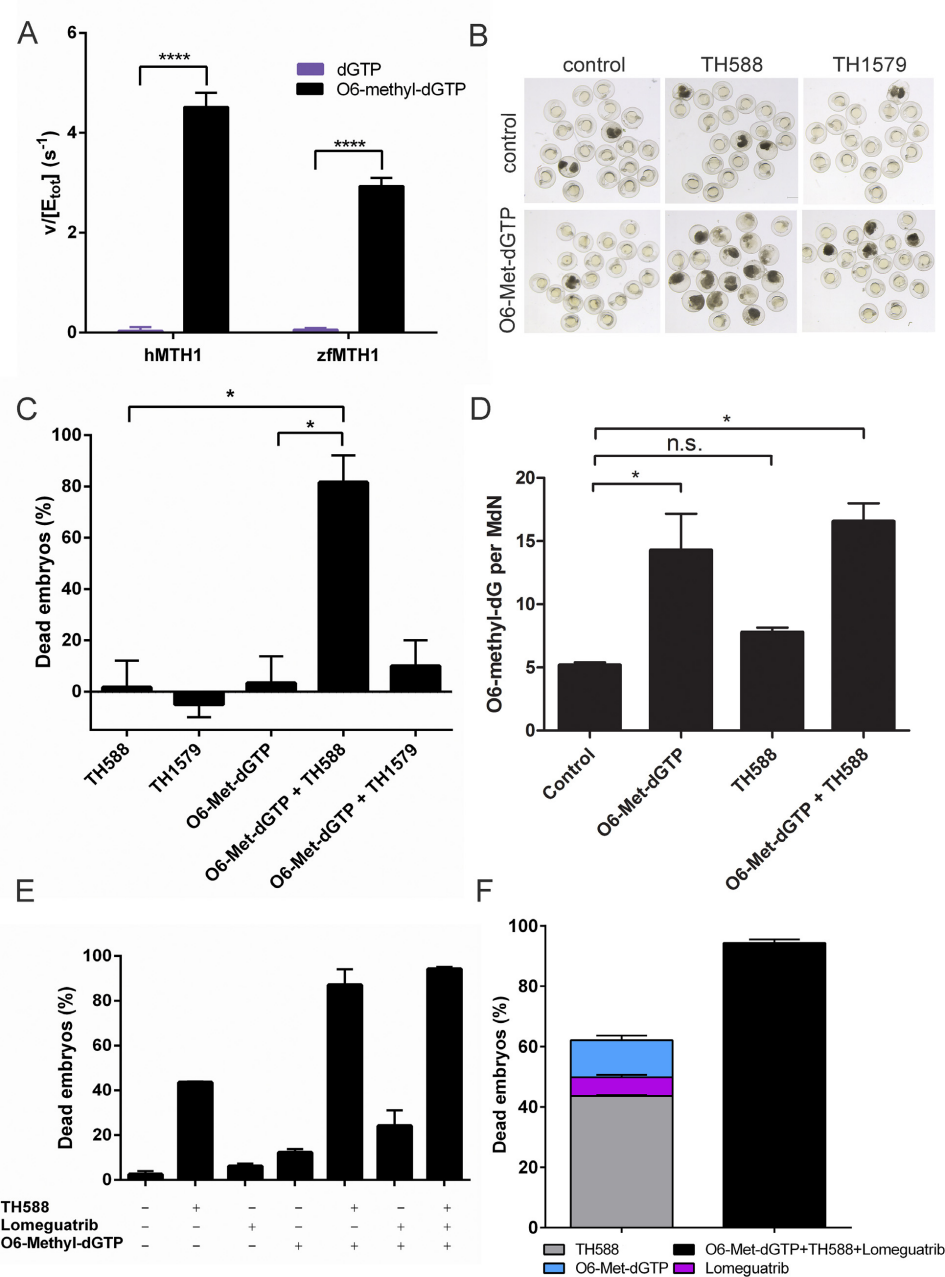
Active zfMTH1 is crucial for zebrafish embryo survival after O6-methyl-dGTP exposure. (**A**) The zfMTH1 enzyme catalyzes the hydrolysis of O6-methyl-dGTP efficiently. 100 μM dGTP or O6-methyl-dGTP was incubated with 5 nM zfMTH1 or hMTH1 for 20 min. Formed PPi was converted to Pi by using an excess of *E. coli* PPase and Pi was detected using malachite green reagent. Statistical significance was determined using multiple comparison and Two way Anova using the GraphPad Prism 6.0 software. (**B**) O6-methyl-dGTP (150 μM) was injected into fertilized zebra fish eggs followed by treatment with DMSO, TH588 (1.5 μM) or TH1579 (1.5 μM). Picture shows zebrafish embryos from a representative experiment. (**C**) Quantification of zebrafish survival. Inhibition of zfMTH1 in combination with microinjecting O6-methyl-dGTP in zebrafish is clearly toxic to fish embryos. Graph shows average and standard deviations from three independent experiments. (**D**) Levels of O6-methyl-dG per million dN in DNA as measured by LC–MS/MS. DNA was extracted from DMSO or TH588 treated zebrafish embryos, zebrafish embryos microinjected with O6-methyl-dGTP or from O6-methyl-dGTP microinjected and TH588 treated zebrafish embryos. Graph shows mean and SEM from two independent experiments. Statistic significance in C and D was tested using multiple comparisons and One way Anova, *P* ≤ 0.05 are indicated by *. (**E**) Percentage dead embryos after microinjection of O6-methyl-dGTP and inhibition of zfMTH1 and MGMT through treatment with TH588 (1.5 μM) and Lomeguatrib (10 μM), alone and in combination, compared to untreated zebrafish embryos. Graph shows average ± SD from three independent experiments. (**F**) Co-treatment of O6-methyl-dGTP injected zebrafish eggs with TH588 and Lomeguatrib significantly decreases the survival of zebrafish embryos compared to the effects of the combined individual treatments.

After showing that MTH1 is an efficient catalyst of O6-methyl-dGTP hydrolysis we decided to investigate the relevance of this MTH1 activity by studying the effect of inactivating MTH1 when exposing to O6-methyl-dGTP in an *in vivo* system. Delivery of nucleotide triphosphates into intact cells is technically challenging due to their hydrophilic properties. The zebrafish embryo however provides a unique *in vivo* model system to study nucleotide triphosphates as those can be microinjected into fertilized eggs from where they distribute equally and ubiquitously throughout the growing embryo. We have previously shown that zebrafish is an excellent *in vivo* model to study the biology of MTH1 (21). The fact that zfMTH1 also displays activity with O6-methyl-dGTP (Figure 4A) prompted us to test the activity of zfMTH1 and human MTH1 with O6-methyl-dGTP also at physiological pH and to compare with the activity towards dGTP. The zfMTH1 enzyme displays considerable activity with O6-methyl-dGTP with a clear preference over dGTP (Figure 6A) although the activity of zfMTH1 with O6-methyl-dGTP is not as high as for hMTH1. We tested TH588 and TH1579, both potent inhibitors of human MTH1 and derived from the same chemical scaffold, for inhibition of zfMTH1. Only TH588 was found to be a potent inhibitor of zfMTH1 (IC_50_ = 12 nM) while no inhibition could be observed with TH1579 even at 10 μM (Supplementary Figure S4A). Based on these results we decided to use zebrafish as a model and to use TH588 and TH1579 as tool compounds with TH1579 acting as a negative control not inhibiting zfMTH1. Microinjecting approximately 2 nl of a 150 μM O6-methyl-dGTP solution itself had no effect on embryo viability (95 ± 7.1% alive, *n* = 60), whereas inhibition of MTH1 using 1.5 μM TH588 in embryos injected with O6-methyl-dGTP reduced viability to 16.7 ± 9.4% (*n* = 60) (Figure 6B and C). Exposure of injected embryos with 1.5 μM of the MTH1 inhibitor TH1579, not targeting zfMTH1, had little effect on viability (88.3 ± 6.2% alive, *n* = 60) (Figure 6B and C). This shows that active zfMTH1 is needed to protect from the toxic effect of O6-methyl-dGTP and shows the importance of MTH1 in removing O6-methyl-dGTP from the nucleotide pool in an *in vivo* model.

### MTH1 protects against O6-methyl-dGTP being incorporated into DNA

To test if O6-methyl-dGTP is incorporated into DNA and exerts its toxicity through induction of DNA damage we extracted DNA from zebrafish embryos microinjected with O6-methyl-dGTP and treated with or without MTH1 inhibitor TH588, as well as from untreated control embryos. Levels of O6-methyl-dG and N7-methyl-dG in DNA were analysed using LC–MS/MS. Results show that embryos microinjected with O6-methyl-dGTP have considerably higher levels of O6-methyl-dG in DNA compared to untreated embryos (Figure 6D). Treatment with TH588 only causes a slightly increased level of O6-methyl-dG in the DNA possibly due to incorporation of endogenously produced O6-methyl-dGTP. We also determined the levels of N7-methyl-dG in DNA as a control and could not observe any significant differences between treatment groups (Supplementary Figure S5). The finding that the levels of N7-methyl-dG in DNA is not affected by MTH1 inhibition is consistent with the observation that N7-methyl-dGTP is not a MTH1 substrate (Supplementary Figure S1B).

O6-methylguanine-DNA methyltransferase (MGMT) is the main repair pathway for repair of O6-methyl-dG in DNA as evidenced by the large impact of cellular MGMT levels on toxicity of alkylating agents (2,42). MGMT removes the methyl-group on O6-guanine when present in DNA (43). We reasoned that inhibition of MGMT would enhance the toxicity of O6-methyl-dGTP injected into fertilized zebrafish embryos only if O6-methyl-dGTP is incorporated into the DNA and therefore analysed the protective role of active MTH1 and MGMT on O6-methyl-dGTP toxicity using TH588 and the MGMT inhibitor Lomeguatrib (44). Analysis of survival of zebrafish embryos injected with O6-methyl-dGTP and treated with the MTH1 inhibitor TH588 and/or the MGMT inhibitor Lomeguatrib shows that neither O6-methyl-dGTP nor Lomeguatrib treatment are toxic on their own. However, inhibition of MGMT causes a 1.3-fold sensitization to O6-methyl-dGTP in absence of TH588 (with active zfMTH1) and a 1.5-fold sensitization to O6-methyl-dGTP in presence of TH588 inhibiting MTH1 compared to the effect of the combined individual treatments 24% vs 18.4% (6.2% + 12.2%) dead embryos and 94.3% versus 62% (6.2% + 12.2% + 43.6%) dead embryos, respectively, consistent with O6-methyl-dGTP being incorporated into the DNA (Figure 6E and F).

### MTH1 deficiency increases apoptosis and decreases viability in Temozolomide treated cells

**Figure 7.**
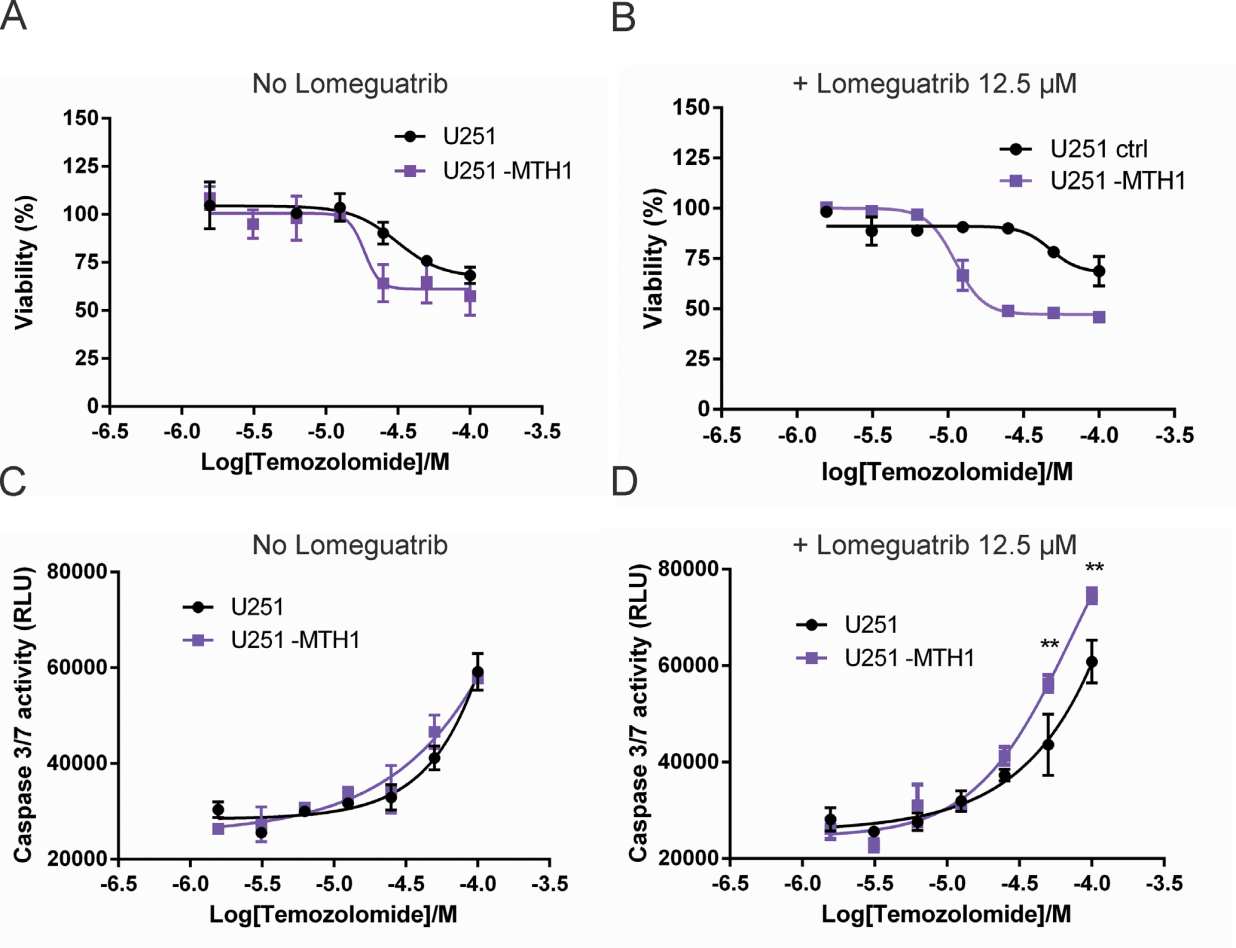
MTH1 deficiency sensitizes cells to Temozolomide. Cell viability of U251 (MTH1 proficient) and U251-MTH1 (MTH1 deficient) was measured using resazurin after 96 h of treatment with Temozolomide and Lomeguatrib. (**A**) EC_50_ values for Temozolomide was determined to 62±4 μM for MTH1 proficient and 17±4 μM for MTH1 deficient cells with active MGMT, respectively. (**B**) EC_50_ values for Temozolomide were 79±2 μM for MTH1 proficient and 11±2 μM for MTH1 deficient cells when MGMT is inhibited with 12.5 μM Lomeguatrib. MTH1 deficiency causes a 3.6-fold sensitization with active MGMT, and increases to 6.6-fold upon MGMT inhibition. Figures show representative experiments out of two independent experiments per treatment with data points in duplicate. Caspase 3/7 activity, detecting early apoptosis, was assayed using U251 and U251-MTH1 cells after treatment with Temozolomide ranging from 0 to 100 μM for 48 h (**C**) or with both Temozolomide and Lomeguatrib (12.5 μM) (**D**). MTH1 deficient cells show increased caspase 3/7 activity at 50 and 100 μM Temozolomide when MGMT is inhibited with 12.5 μM Lomeguatrib. Shown are representative experiments out of three independent experiments with data points in duplicate. Data are presented as average ± SEM. Statistical significant differences were determined using multiple comparison and Two way Anova in GraphPad Prism 6.0, *P* ≤ 0.01 is indicated by**.

Treatment of the glioblastoma cell line U251-MTH1 (MTH1 deficient) and the isogenic U251 cell line (MTH1 proficient) (Supplementary Figure S6) with Temozolomide shows that the cells lacking MTH1 are more sensitive to Temozolomide as assessed by viability assay using resazurin (Figure 7A) with EC_50_ values 3.6-fold higher for the MTH1 proficient cell line (62±4 μM) compared to the MTH1 deficient cell line (17±10 μM). The difference in sensitivity to Temozolomide between the two cell lines increases to 6.6-fold when MGMT is inhibited using 12.5 μM Lomeguatrib (EC_50_ = 79±2 μM for the MTH1 proficient cell line and EC_50_ = 11±2 μM for the MTH1 deficient cell line) (Figure 7A and B). Inhibition of MGMT using Lomeguatrib (12.5 μM) also causes increased early apoptosis as measured by Caspase 3/7 activity in the MTH1 deficient (U251-MTH1) compared to the MTH1 proficient cell line (U251) (Figure 7C and D). This result is consistent with what would be expected if MTH1 protects the cell by hydrolyzing O6-methyl-dGTP and thereby preventing its incorporation into DNA. Higher levels of O6-methyl-dG in the DNA through incorporation of O6-methyl-dGTP would make the cell more dependent on functional MGMT for cell survival.

## DISCUSSION

### Activity of MTH1 with O6-methyl-GTP

MTH1 is here shown to efficiently catalyze the hydrolysis of O6-methyl-dGTP but was also shown to display low activity with O6-methyl-GTP (Figure 1B, Table 1). However, since the concentration of GTP in the cell is approximately 100 times higher than the concentration of dGTP (45) the concentration of O6-Methyl-GTP is likely to be magnitudes higher than O6-Methyl-dGTP and the activity of MTH1 may also be relevant and important to prevent incorporation into RNA, which has been shown to change the fidelity and to decrease the translation rate (15). MTH1 has been reported to display an approximately 20-fold preference for 8-oxo-dGTP over 8-oxo-GTP (46) mostly due to a higher *K*_m_ value for the ribonucleotide substrate. For O6-methyl-dGTP and O6-methyl-GTP there is a large difference between both the *K*_m_ and the *k*_cat_ values (Table 1) indicating that both affinity and catalytic activity is affected by reducing the 2′ carbon as in the O6-methylated guanine ribonucleotide. Analysis of the role of MTH1 on removal of O6-methyl-GTP from the nucleotide pool will be the subject of future research.

### Activity of NUDT17 and NUDT18 with O6-methyl-dGTP

NUDT17 and NUDT18 were the only two enzymes apart from MTH1 among the tested NUDIX hydrolases that displayed O6-methyl-dGTP activity. These enzymes have previously been shown to display activity with oxidized nucleotides like MTH1, but with considerably lower activity (41). This study further supports the notion that NUDT17 and NUDT18 share some features with MTH1. NUDT17 also seems to display some preference for methylated nucleotides over nonmethylated nucleotides similar to MTH1, as observed when assessing activity with a larger panel of methylated and nonmethylated nucleotides (Figure 3B). However, the activity of NUDT17 and NUDT18 is ∼80-fold lower with O6-methyl-dGTP compared to MTH1 and is not likely to make any major contribution to the hydrolysis of O6-methyl-dGTP in MTH1 proficient cells.

### Analysis of MTH1 structures with bound O6-methyl-dGMP

We sought to provide an explanation to the high activity of MTH1 with O6-methyl-dGTP by analysing the structures of both human and zebrafish MTH1 in complex with O6-methyl-dGMP. O6-methyl-dGMP was found to be clearly bound to both human and zebrafish MTH1 as seen from the electron density in Figure 5A and B. Given the shift of 1.1 Å in position of the base of O6-methyl-guanine compared to when 8-oxo-dGMP is bound to MTH1 (see Figure 5C), it is likely that the methylation of O6 is strengthening the binding of the base to the hydrophobic pocket. The residues Glu52, Glu56 and Glu100 are expected to bind the magnesium needed for hydrolysis, and as shown in Figure 5C their positions are identical. It is clear that the hydrolysis of the phosphates is performed in the same manner for 8-oxo-dGTP and O6-methyl-dGTP and the kinetics of the MTH1 activity is determined by the binding of the substrate to the active site. The hydrophobic pocket has previously been explored for drug development as described for TH287 and TH588 (22). The structures are highly similar as shown by RMSD values, and the main difference is the position of the methylated base. The structural analysis reveals differences in the hydrogen bonding network when human MTH1 binds 8-oxo-dGMP and O6-methyl-dGMP as shown in Figure 5C. Interestingly, when binding O6-methyl-dGMP Asp119 needs to be protonated but when binding 8-oxo-dGMP it seems as if either Asp119 or Asp120 could be protonated (17) and in the MTH1 structure with 8-oxo-dGTP bound Asp119 is protonated (47). The few differences in the binding pocket, Y7L and P118A going from human to zebrafish, do not seem to influence the binding of O6-methyl-dGMP as shown in Supplementary Figure S4B. The observed binding of calcium ions in zfMTH1 is likely due to the presence of calcium in the successful crystallization conditions. Calcium ions were found to bind in the crystal contact with another protein monomer, however, the metal ions also bind in to the NUDIX-box motif as expected (Supplementary Figure S7) as previously shown for the related protein NUDT15 (41). The binding mode is however different between zfMTH1 and NUDT15 (Supplementary Figure S8) and based on the available data the position of the magnesium ions in zfMTH1 in solution remains unknown. Another explanation for calcium ions being found in the zfMTH1 structure could be that zfMTH1 utilizes Ca^2+^ instead of Mg^2+^. However, when testing the activity of zfMTH1 upon replacing Mg^2+^ with Ca^2+^ no activity was observed disproving this theory (Supplementary Figure S9).

### Physiological relevance of the MTH1 activity with O6-methyl-dGTP

We here present O6-methyl-dGTP as a novel and excellent substrate for MTH1. MTH1 displays a catalytic efficiency with O6-methyl-dGTP that is in the same range as for its known substrate 8-oxo-dGTP, implying that hydrolysis of O6-methyl-dGTP indeed is an important function of MTH1. This activity was found to be conserved in MTH1 homologues from several different species, ranging from organisms as distantly related as plants and zebrafish to humans, strongly indicating that this activity is beneficial to the cell. O6-methyl-G adducts are removed from DNA by MGMT in a suicide reaction where the methyl group is transferred to a cysteine in the active site of the enzyme (14). Mammalian cells lacking MGMT have been shown to be very sensitive to methylating agents (48), and mice lacking MGMT are hypersensitive to agents that produce O6-methyl-G (49,50). If not removed O6-methyl-G in DNA leads to mutations, DNA strand breaks and collapse of replication forks (5). Removal of O6-methyl-dGTP from the nucleotide pool would potentially be of high importance to prevent incorporation into DNA in cells where the capacity of MGMT is low and when the cell is exposed to methylating substances resulting in levels of O6-methyl-G in the DNA high enough to override the capacity of MGMT. Some tumors lack MGMT due to epigenetic silencing of the MGMT gene and consequently such tumors are more sensitive to methylating agents and are reported to respond better to treatment with alkylating drugs (51). Inhibition of MTH1 in such a background may sensitize the tumors even more to methylating agents and further improve the response. We show that zebrafish embryos are dependent on MTH1 activity for survival when exposed to O6-methyl-dGTP. We demonstrate that O6-methyl-dGTP is incorporated into DNA and that the toxicity of O6-methyl-dGTP is enhanced when MGMT is inhibited. Together, these results strongly suggest that MTH1 catalyzed hydrolysis of O6-methyl-dGTP to O6-methyl-dGMP renders the methylated nucleotide harmless by preventing its incorporation into DNA.

Temozolomide is an alkylating drug known to exert its toxicity through production of O6-methyl-dG (1). Temozolomide is used for treatment of glioblastoma multiforme since it due to its small size and lipophilicity is among the few drugs that can pass the blood brain barrier and can therefore be used to treat cancer in the central nervous system (33). Temozolomide is a prodrug that needs to undergo hydrolysis to form the active metabolite 3-methyl-(triazen-1-yl)imidazole-4-carboxamide that in turn breaks down to produce the reactive methyldiazonium ion metabolite (52,53). Although no study yet has reported the formation of O6-methyl-dGTP in cells upon Temozolomide treatment, O6-methyl-dGTP was shown to be produced in cells treated with N-methyl-N-nitrosourea that decomposes to the same reactive methyldiazonium ion metabolite as Temozolomide (3). This strongly suggests that O6-methyl-dGTP also is formed in cells upon Temozolomide treatment. This notion is further strengthened by the fact that glioblastoma cells deficient in MTH1 were shown to display a higher sensitivity to Temozolomide compared to MTH1 proficient cells, most likely through different abilities to remove formed O6-methyl-dGTP. Since Temozolomide is more effective in treating cancers with low MGMT levels we tested the effect of co-treatment with the MGMT inhibitor Lomeguatrib (Figure 7B and D). Inhibition of MGMT was found to increase the observed difference in Temozolomide sensitivity between MTH1 deficient and MTH1 proficient cells clearly supporting a role of MTH1 in protecting against the toxic effects of O6-methyl-dGTP, formed upon methylation of dGTP, also in human cells. MTH1 inhibitors could potentially be used to potentiate Temozolomide and other alkylating drugs, acting through O6-methylation of guanine. The shift in sensitivity to Temozolomide between the MTH1 proficient and the MTH1 deficient cells (Figure 7A and B) can be ascribed to the fraction of Temozolomide toxicity that is exerted through methylation of dGTP in the free nucleotide pool producing O6-methyl-dGTP. Our result also shows that eukaryotic DNA polymerases can incorporate O6-methyl-dGTP into the growing DNA chain during replication as evidenced by the results of our analysis of zebrafish DNA (Figure 6D) showing increased levels of O6-methyl-dG after microinjection of O6-methyl-dGTP together with the experiments with human cells demonstrating that MTH1 deficiency increases the sensitivity to Temozolomide treatment (Figure 7). This has to our knowledge previously only been shown for T4 bacteriophage DNA polymerase (54,55).

We observed early apoptosis (Figure 7C and D) after 48 h of Temozolomide treatment consistent with published data describing that the Temozolomide toxicity appears at the second round of replication after Temozolomide exposure due to futile mismatch repair that induces apoptosis (56).

The major endogenous methylator SAM was reported to function as a weak alkylating substance and was found to produce methylated nucleotides in the DNA in the form of N7-methylguanine and N3-methyladenine but also O6-methylguanine (2) indicating that there also may be a need to remove O6-methyl-dGTP under normal cellular conditions. The evolutionarily conserved MTH1 activity with O6-methyl-dGTP presented here, as well as the presence of MGMT enzymes in both prokaryotes and eukaryotes, together removing O6-methylated guanines from the DNA and the free nucleotide pool clearly marks the importance of protecting the cell against alkylating species producing O6-methyl-dG.

In conclusion, this study reveals a novel function of MTH1 as constituting another line of defence against the cytotoxic and mutagenic O6-methylation of guanine by efficiently catalysing the hydrolysis of O6-methyl-dGTP and thereby preventing its incorporation into DNA. MTH1 emerges as a multifunctional enzyme important for sanitizing the cell from damaged nucleotides.

## DATA AVAILABILITY

Atomic coordinates and structure factors for the reported crystal structures have been deposited with the Protein Data Bank under accession numbers 5OTM (hMTH1) and 5OTN (zfMTH1).

## Supplementary Material

Supplementary Data
