# Supplementary material for: MutT homologue 1 (MTH1) catalyzes the hydrolysis of mutagenic O6-methyl-dGTP
Source: Nucleic Acids Res. 2018 Oct 10;46(20):10888–904. doi: 10.1093/nar/gky896 (PMC6237811; doi:10.1093/nar/gky896)
Supplement: Supplementary Data [file gky896_supplemental_files.pdf]

SUPPLEMENTARY TABLES AND FIGURES

Supplementary Table S1: Data collection statistics

Data collection	hMTH1	zfMTH1
**PDB code**	5OTM	5OTN
**Space group**	$P2_12_12_1$	$P2_12_12_1$
**Cell dimensions**		
a, b, c **(Å)**	59.6 67.2 79.0	44.5 55.4 65.9
α, β, γ **(°)**	90, 90, 90	90, 90, 90
**Resolution (Å)**	47.57–1.80 (1.84–1.80)	28.32–0.99 (1.01–0.99)
**R_{merge} (%)**	8.5 (82.3)	6.3 (93.0)
**I/σ (I)**	13.7 (2.1)	14.1 (1.3)
**Completeness (%)**	98.9 (97.7)	99.2 (94.9)
**CC(1/2) (%)**	99.9 (75.6)	99.9 (41.8)
**Redundancy**	4.5 (4.5)	6.3 (3.7)
**Refinement**		
**Resolution (Å)**	47.57–1.80	28.32–0.99
**No. unique reflections**	29598 (1705)	90549 (4198)
**R_{work}/R_{free} ***	19.6/24.7	12.1/13.3
**No. atoms**		
**Protein**	2564	1408
**Compound**	48	24
**Other ligands**	44	70
**Water**	242	163
**B-factors**		
**Protein**	24.379	10.224
**Compound**	23.277	6.826
**Other ligands**	46.111	19.948
**Water**	28.603	25.374
**R.m.s. deviations**		
**Bond lengths (Å)**	0.0078	0.0138
**Bond angles (°)**	1.326	1.629
**Ramachandran plot, residues in (%)**		
**Most favourable region**	99.35	99.35
**Additional allowed region**	0.65	0.65

Highest resolution shell is shown in parenthesis
* For PDB code 5OTN (zfMTH1), 2.5% of all reflections are set aside for R_{free} calculations instead of the 5% standard.

Supplementary Figure S1

A

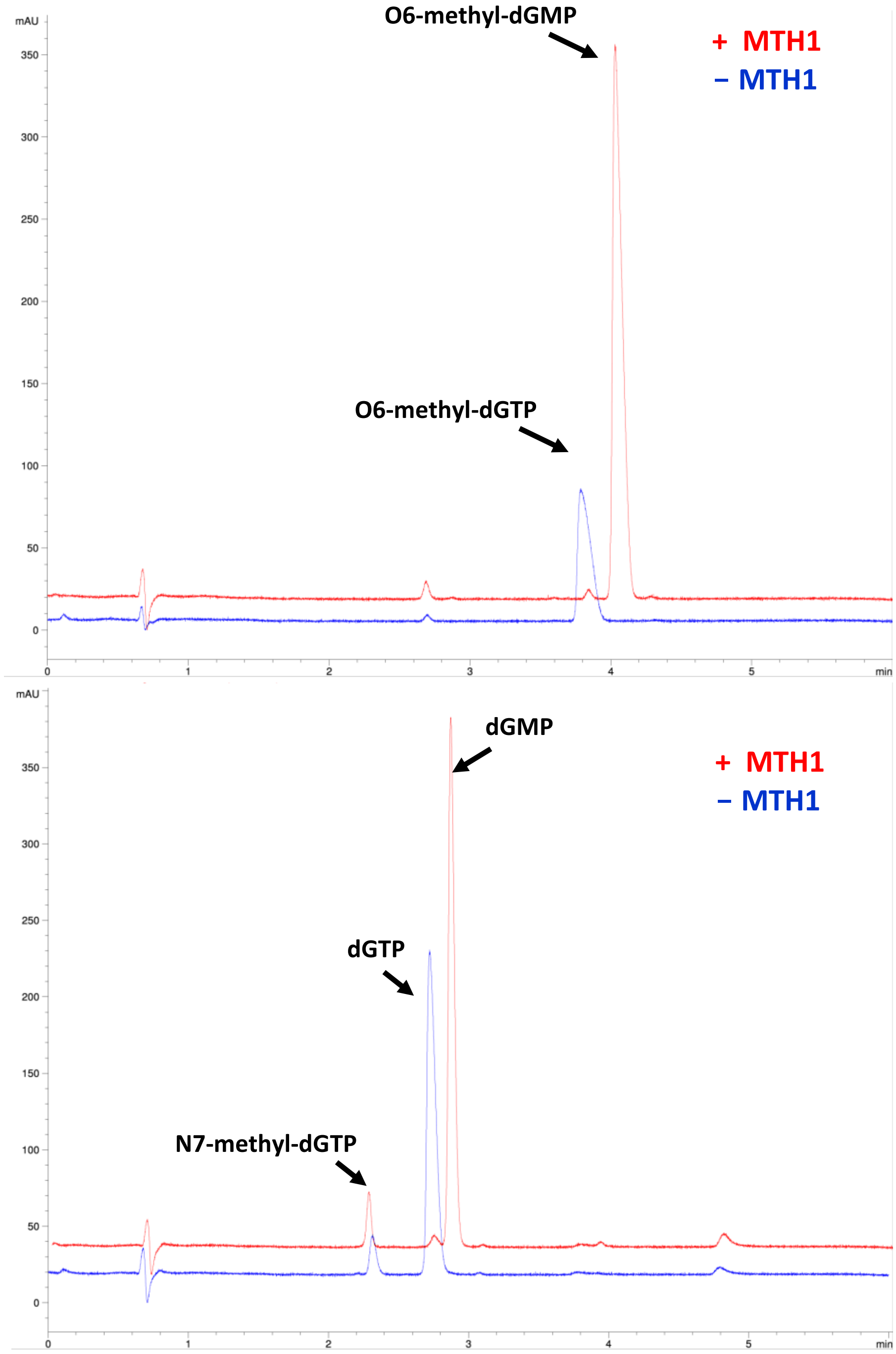

Supplementary Figure S1. (A) HPLC analysis of reaction products of O6-methyl-dGTP (500 µM) after incubation with MTH1 (250 nM) (red) or without MTH1 (blue) in MTH1 reaction buffer pH 8.0, showing that MTH1 catalyses the hydrolysis of O6-methyl-dGTP to O6-methyl-dGMP. (**B)** HPLC analysis of N7-methyl-dGTP preparation (500 µM dGTP incubated with 1000 µM MMS for 20h at 22°C) after incubation with MTH1 (250 nM) (red) or without MTH1 (blue) in MTH1 reaction buffer pH 8.0 for 10 min at 22°C, showing no detectable MTH1 catalysed hydrolysis of N7-methyl-dGTP. Instead, pronounced MTH1 dependent hydrolysis of dGTP to dGMP is observed.

Supplementary Figure S2

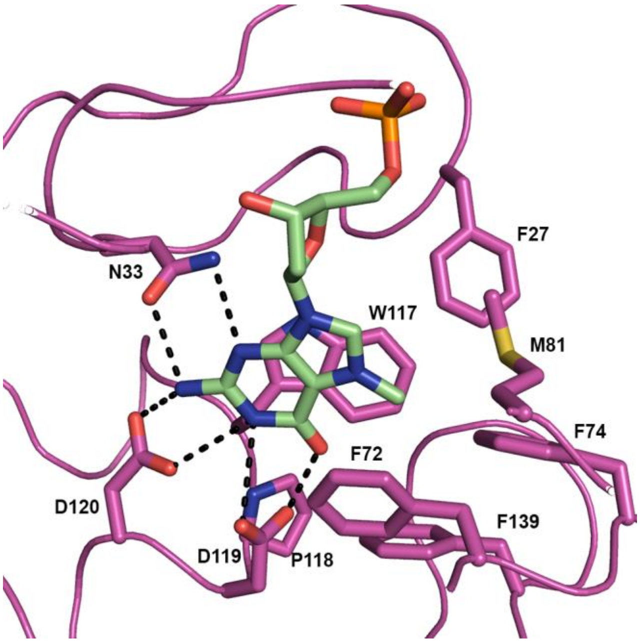

Supplementary Figure S2. N7-methyl-dGTP (green sticks) docked *in silico* into the active site of MTH1 (magenta) showing a good fit. Residues important for binding and residues of the hydrophobic pocket are shown as sticks and are labelled.

Supplementary Figure S3

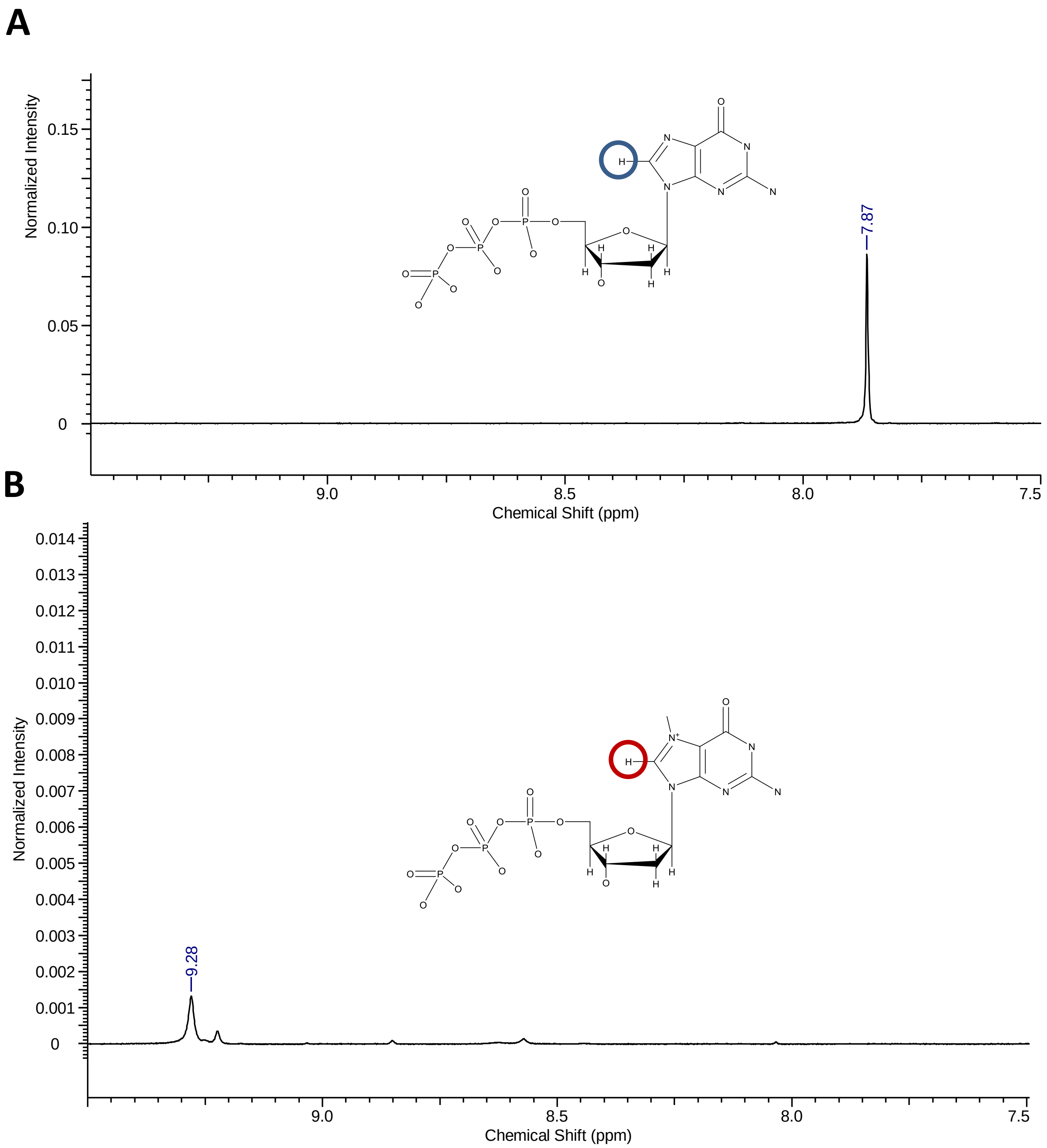

Supplementary Figure S3. (A) NMR analysis of H8 (encircled in blue) of dGTP (10 mM) in 3:1 DMSO-d6:D_2O. (**B**) NMR analysis of H8 (encircled in red) of N7-methyl-dGTP (produced by incubation of 10 mM dGTP overnight with 300 mM methyl iodide) in 3:1 DMSO-d6:D_2O. The methylation on N7 of dGTP generates a large deshielding effect of H8 causing a delta shift of 1.41 ppm.

Supplementary Figure S4

A

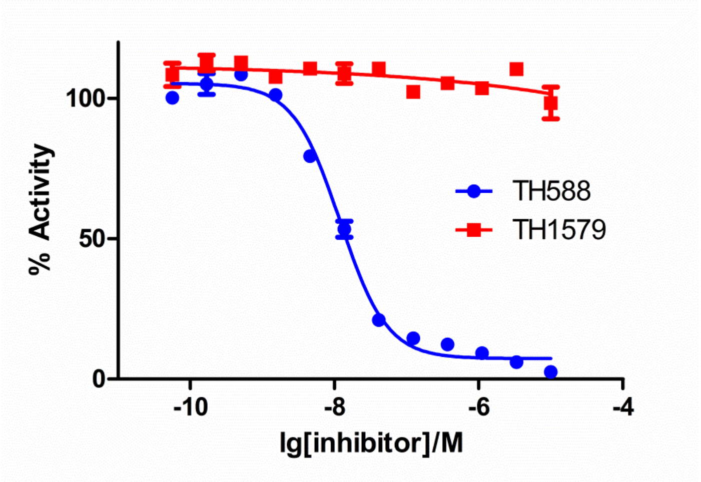

B

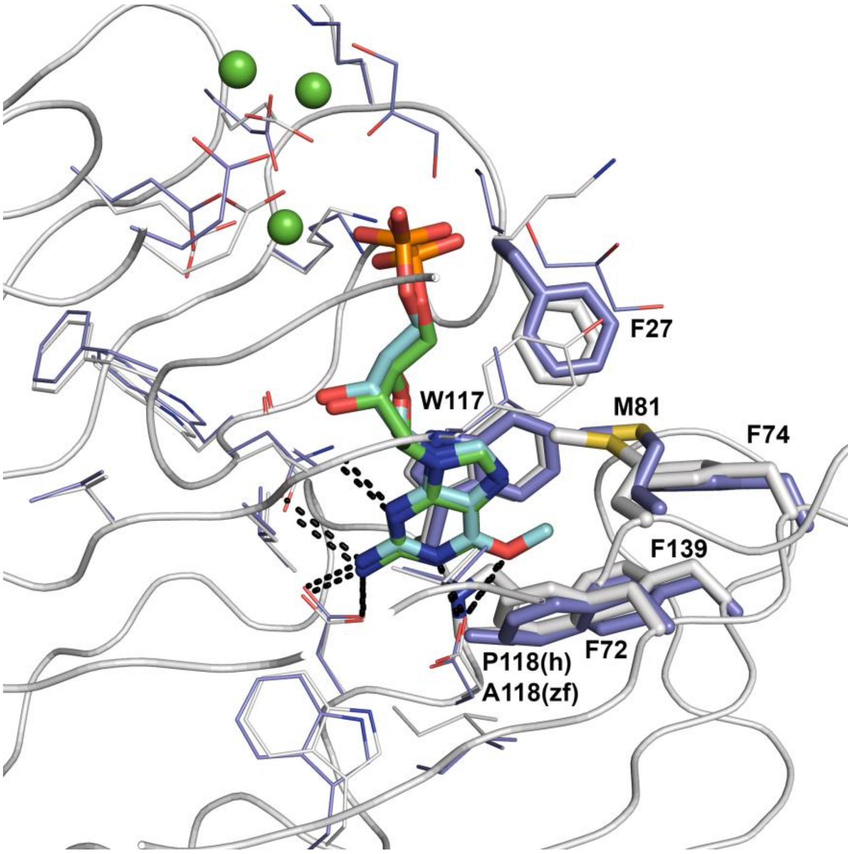

Supplementary Figure S4. (**A)** Human MTH1 inhibitor TH588 but not TH1579 inhibits zfMTH1. Dose response curves were run using 12 inhibitor concentrations ranging from 10 µM to 0.06 nM. Data points were recorded in duplicate. IC_{50} value of zfMTH1 for TH588 were determined to 12 nM using the GraphPad Prism software. (**B)** Comparison of structures of O6-methyl-dGMP bound to human and zebrafish MTH1. Human MTH1 is shown in off-white and zebrafish MTH1 is shown in purple. O6-methyl-dGMP is shown in green when bound to human MTH1 and in cyan when bound to zebrafish MTH1. Residues of the hydrophobic pocket are shown as sticks and are labelled. Other residues within 6Å of O6-methyl-dGMP are shown as lines. Calcium-ions bound in the zebrafish MTH1 structure are shown as green spheres.

Supplementary Figure S5

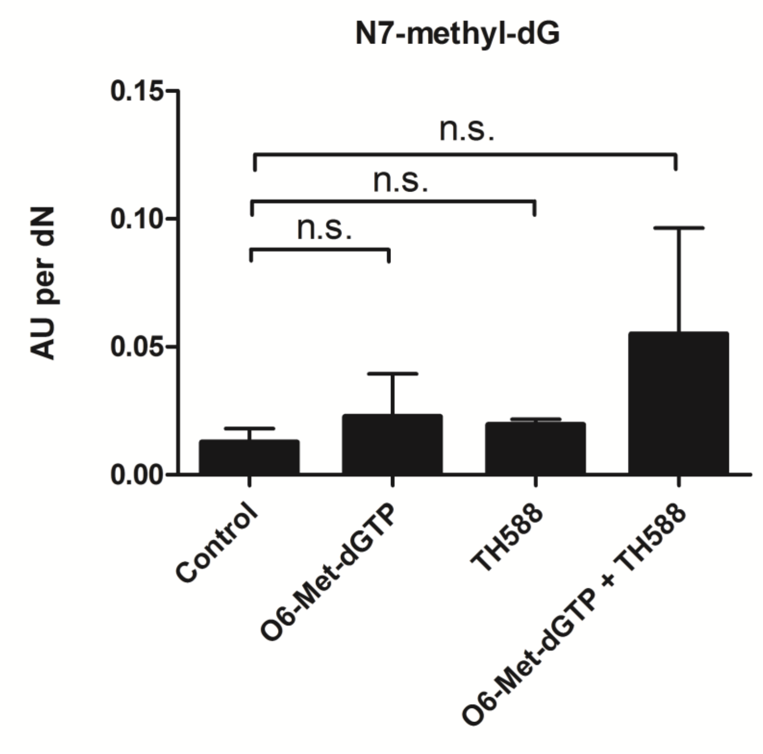

Supplementary Figure S5. N7-Methyl-dG levels in DNA extracted from zebrafish as analysed using LC-MS/MS. Bars represent average ± SEM from two independent experiments. Statistical significance was tested using multiple comparison and One way Anova using the GraphPad Prism software.

Supplementary Figure S6

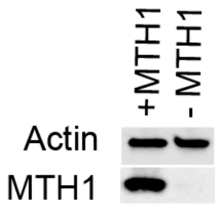

Supplementary Figure S6. Western blot analysis of lysate from human glioblastoma cell line U251, proficient in MTH1 (labelled +MTH1), and U251-MTH1 in which MTH1 was removed using CRISPR/Cas9 (labelled -MTH1).

Supplementary Figure S7

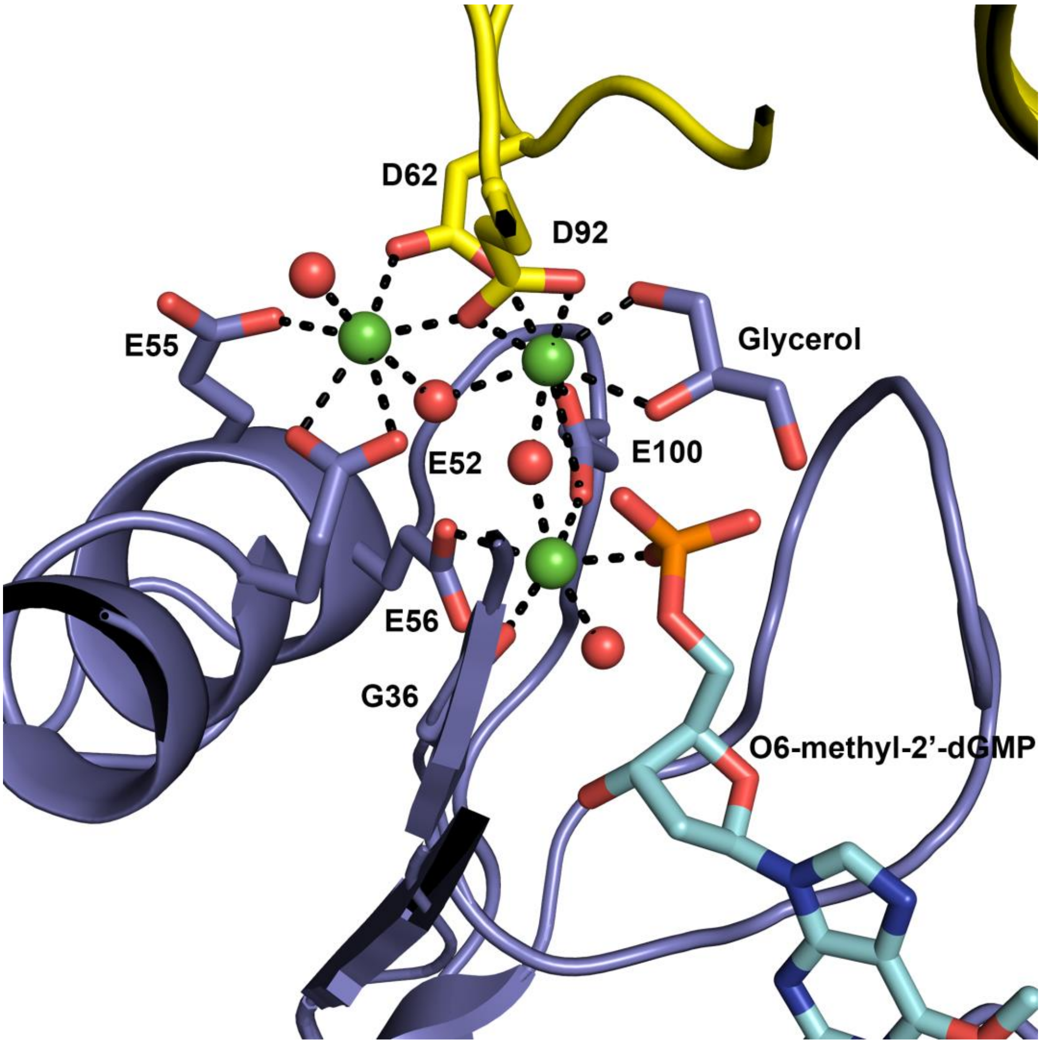

Supplementary Figure S7. Binding of calcium-ions in the zebrafish MTH1 structure. Calcium-ions are shown as green spheres and water molecules as red spheres. The crystallographic symmetry neighbour involved in calcium binding is shown in yellow. Important binding residues are shown as sticks and are labelled.

Supplementary Figure S8

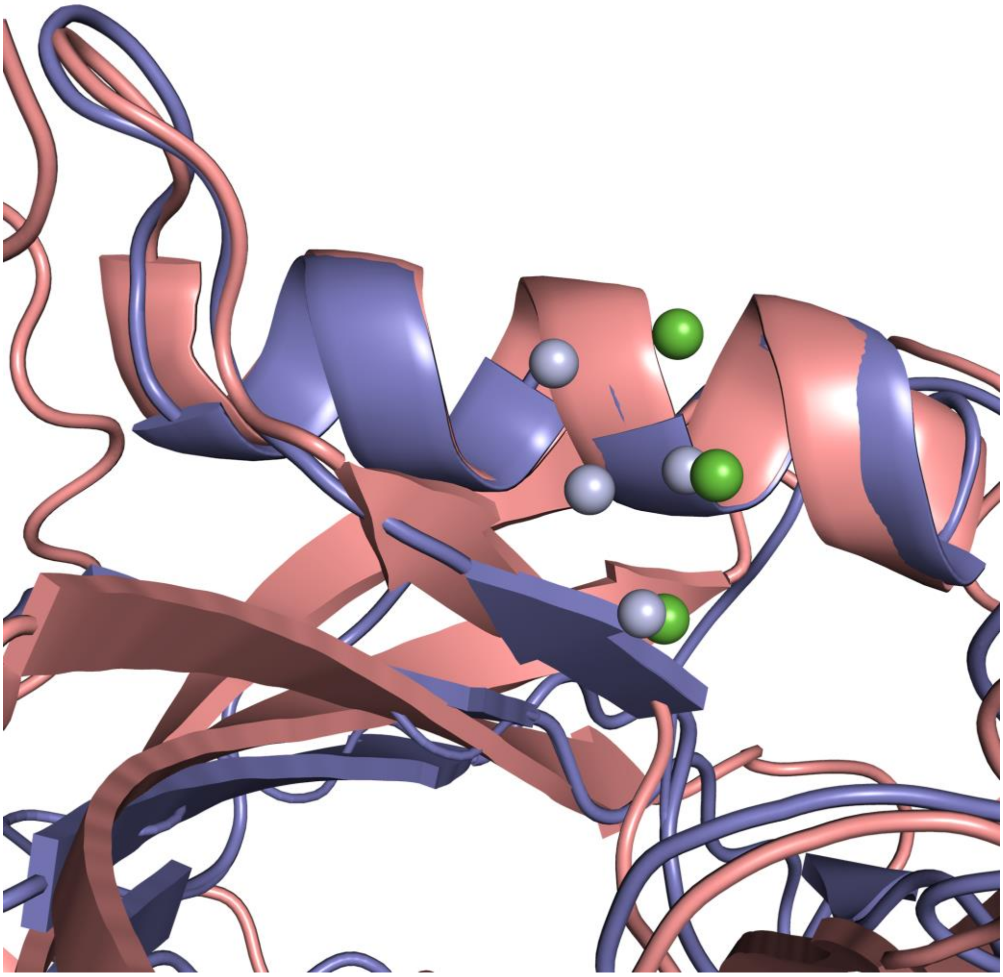

Supplementary Figure S8: Comparison of metal binding in zebrafish MTH1 and human NUDT15 (MTH2, pdb 5BON). Zebrafish MTH1 is shown in purple and human NUDT15 in dark pink. Picture is zoomed in on the NUDIX-box (central α-helix and β-strand, which is the metal binding motif. Magnesium-ions in NUDT15 are shown as light blue spheres and calcium-ions bound to zebrafish MTH1 are shown as green spheres.

Supplementary Figure S9

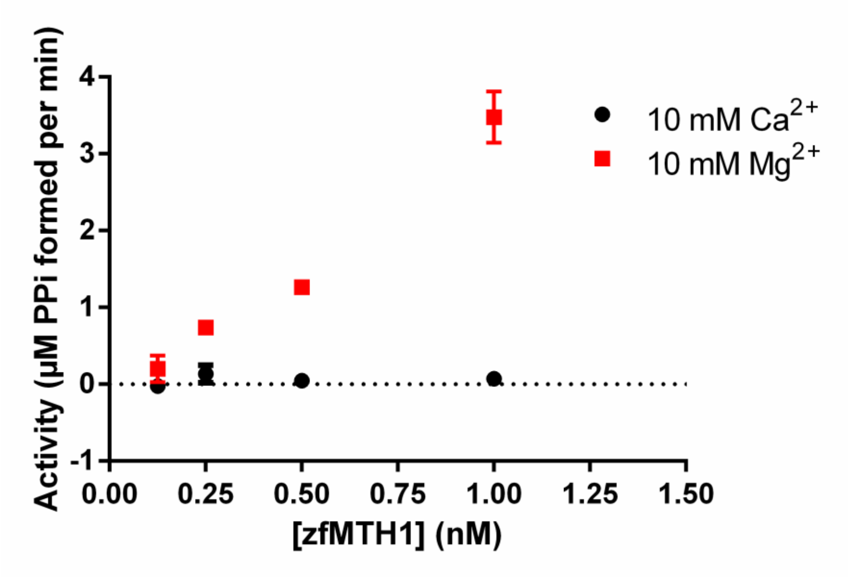

Supplementary Figure S9. Ca^{2+} cannot replace Mg^{2+} for zfMTH1 activity. Activity of zfMTH1 with 100 µM dGTP was tested at 4 different concentrations of zfMTH1 in presence of 10 mM Ca^{2+} or 10 mM Mg^{2+} for 20 minutes. Formed PPi was detected using PPiLight inorganic pyrophosphate assay (Lonza).
